# Crystal structures of three indole derivatives: 3-ethnyl-2-methyl-1-phenyl­sulfonyl-1*H*-indole, 4-phenyl­sulfonyl-3*H*,4*H*-cyclo­penta­[*b*]indol-1(2*H*)-one and 1-{2-[(*E*)-2-(5-chloro-2-nitro­phen­yl)ethen­yl]-1-phenyl­sulfonyl-1*H*-indol-3-yl}ethan-1-one chloro­form monosolvate

**DOI:** 10.1107/S2056989015014917

**Published:** 2015-08-15

**Authors:** S. Gopinath, K. Sethusankar, Bose Muthu Ramalingam, Arasambattu K. Mohanakrishnan

**Affiliations:** a Department of Physics, RKM Vivekananda College (Autonomous), Chennai 600 004, India; b Department of Organic Chemistry, University of Madras, Guindy Campus, Chennai 600 025, India

**Keywords:** crystal structure, phenyl­sulfon­yl, indole derivatives, hydrogen bonding

## Abstract

The title compounds, C_17_H_13_NO_2_S, (I), C_17_H_13_NO_3_S, (II), and C_24_H_17_ClN_2_O_5_S·CHCl_3_, (III), are indole derivatives. Compounds (I) and (II) crystalize with two independent mol­ecules in the asymmetric unit. The indole ring systems in all three structures deviate only slightly from planarity, with dihedral angles between the planes of the pyrrole and benzene rings spanning the tight range 0.20 (9)–1.65 (9)°. These indole ring systems, in turn, are almost orthogonal to the phenyl­sulfonyl rings [range of dihedral angles between mean planes = 77.21 (8)–89.26 (8)°]. In the three compounds, the mol­ecular structure is stabilized by intra­molecular C—H⋯O hydrogen bonds, generating *S*(6) ring motifs with the sulfone O atom. In compounds (I) and (II), the two independent mol­ecules are linked by C—H⋯O hydrogen bonds and C—H⋯π inter­actions, while in compound (III), the mol­ecules are linked by C—H⋯O hydrogen bonds, generating *R*_2_^2^(22) inversion dimers.

## Abstract 2

The title compounds, (I), (II) and (III), are indole derivatives. Compounds (I) and (II) present two independent moieties in the asymmetric unit, and their packing is led by C—H⋯O hydrogen bonds and C—H⋯π inter­actions. In compound (III), the C—H⋯O hydrogen bonds form 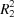
(22) inversion dimers.

## Chemical context   

Indole is an aromatic heterocyclic group, the parent of a large number of important compounds in nature with significant biological activity (Kaushik *et al.*, 2013 ▸). The indole ring system occurs in plants (Nigovic *et al.*, 2000 ▸); for example, indole-3-acetic acid is a naturally occuring auxin that controls several plant growth activities (Moore, 1989 ▸; Fargasova, 1994 ▸). Indole derivatives exhibit anti­bacterial, anti­fungal (Singh *et al.*, 2000 ▸), anti­tumor (Andreani *et al.*, 2001 ▸), anti­hepatitis B virus (Chai *et al.*, 2006 ▸) and anti-inflammatory (Rodriguez *et al.*, 1985 ▸) activities. They are also used as bioactive drugs (Stevenson *et al.*, 2000 ▸) and have also been proven to display high aldose reductase inhibitory (Rajeswaran *et al.*, 1999 ▸) and anti­microbial activities (Amal Raj *et al.*, 2003 ▸). Indole derivatives are also found to possess hypertensive, muscle relaxant (Hendi & Basangoudar, 1981 ▸) and anti­viral (Kolocouris *et al.*, 1994 ▸) activities. Some of the indole alkaloids extracted from plants possess inter­esting cytotoxic and anti­parasitic properties (Quetin-Leclercq, 1994 ▸). Against this background, the X-ray structure determination of 3-ethnyl-2-methyl-1-phenyl­sulfonyl-1*H*-indole, (I), 4-phenyl­sulfonyl-3*H*,4*H*-cyclo­penta­[*b*]indol-1(2*H*)-one, (II), and 1-{2-[(*E*)-2-(5-chloro-2-nitro­phen­yl)ethen­yl]-1-phenyl­sulfonyl-1*H*-indol-3-yl}ethan-1-one chloro­form monosolvate, (III), has been carried out to study their structural aspects and the results are presented here.
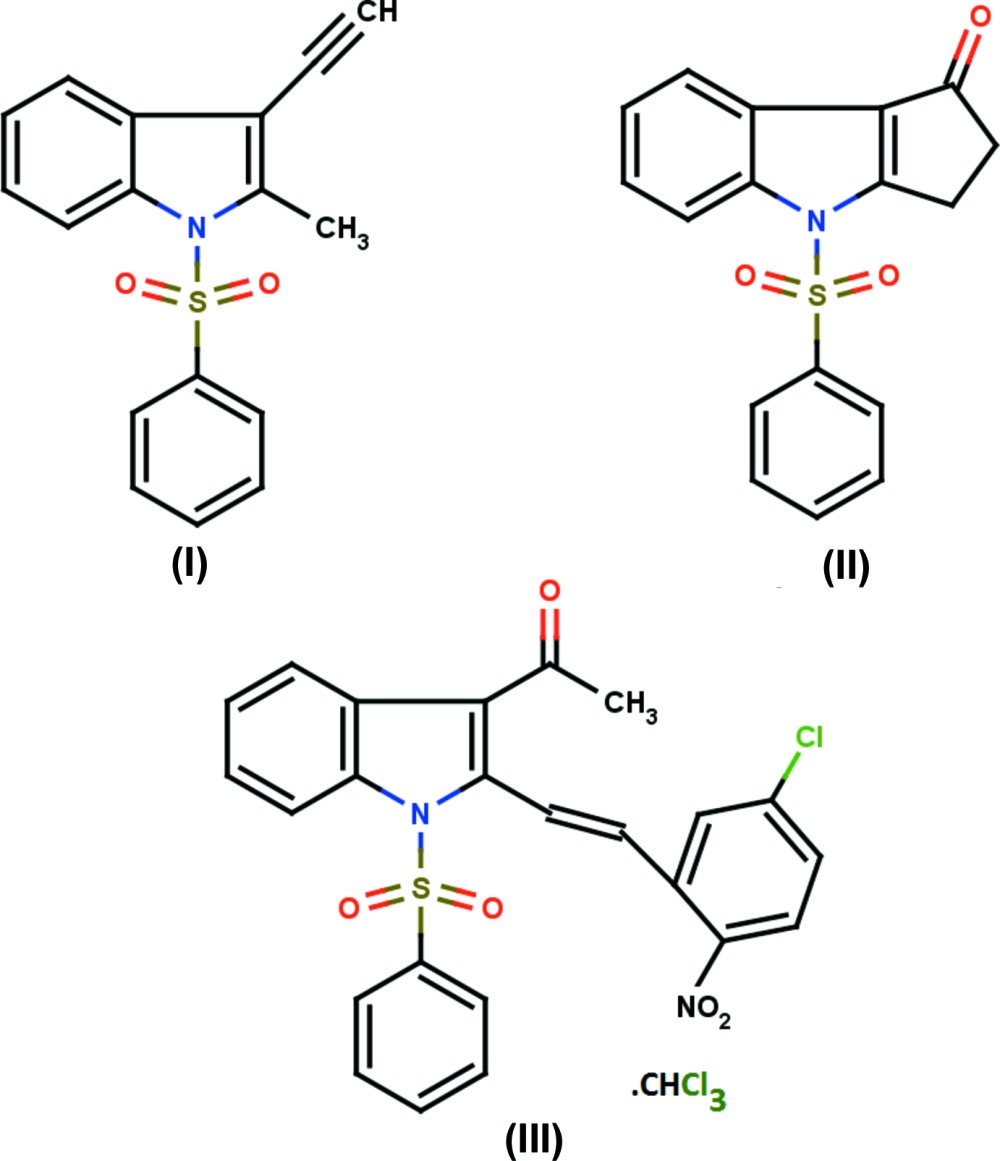


## Structural commentary   

**Figure 1:**
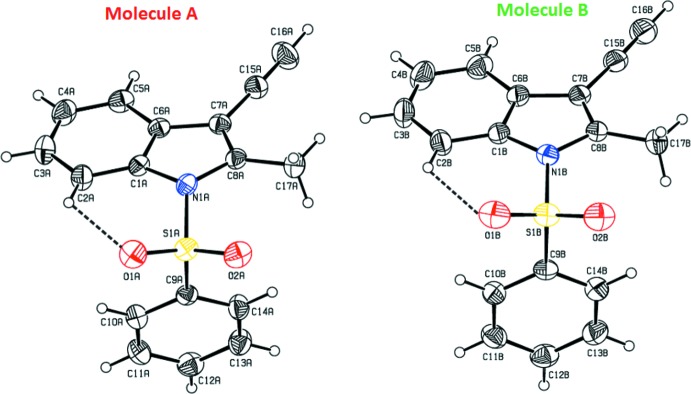
The mol­ecular structure of the compound (I), showing the atom-numbering scheme. The intra­molecular C2*A*—H2*A*⋯O2*A* and C2*B*—H2*B*⋯O2*B* inter­actions (mol­ecules *A* and *B*), which generate two *S*(6) ring motifs, are shown as dashed lines. Displacement ellipsoids are drawn at the 30% probability level.

**Figure 4:**
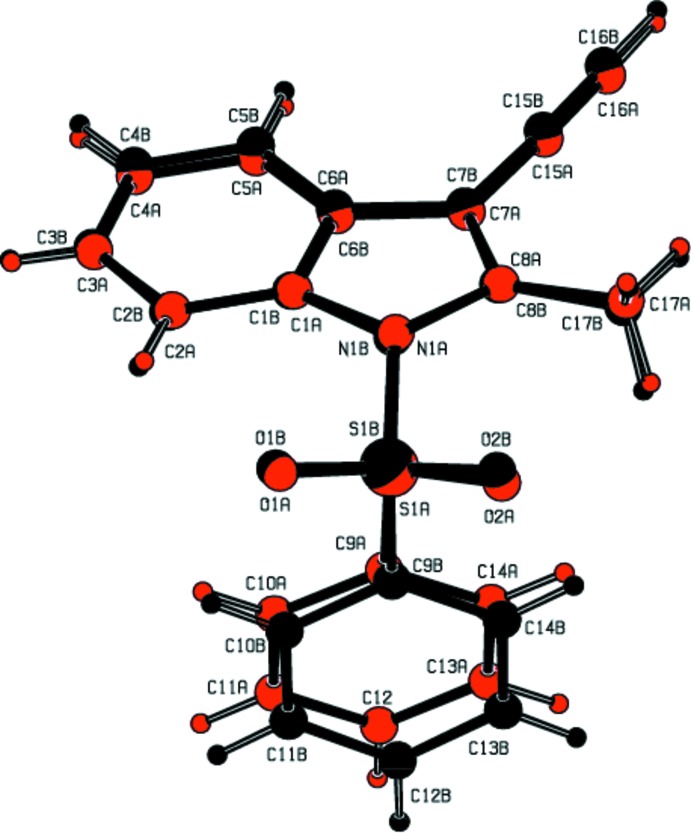
Mol­ecules *A* (red) and mol­ecule *B* (black) of title compound (I) overlapping with each other. H atoms are shown as spheres of arbitrary radius.

The mol­ecular structures of title compounds (I), (II) and (III) are shown in Figs. 1 ▸, 2 ▸ and 3 ▸, respectively. Compounds (I) and (II) comprise two crystallographically independent mol­ecules (*A* and *B*) in the asymmetric unit. The corresponding bond lengths and bond angles of mol­ecules *A* and *B* [in compounds (I) and (II)] agree well with each other, as illustrated in Figs. 4 ▸ and 5 ▸. The indole ring systems depart slightly from planarity, the dihedral angles formed between the pyrrole rings and benzene rings being 1.65 (9) and 0.97 (10) [mol­ecules *A* and *B* of compound (I)], 0.20 (9) and 0.86 (9) [mol­ecules *A* and *B* of compound (II)], and 1.34 (14)° [compound (III)].

The indole ring systems are almost orthogonal to the phenyl­sulfonyl rings [dihedral angles = 77.21 (8) and 89.26 (8)° in (I), 78.98 (7) and 80.48 (8)° in (II), and 83.17 (13)° in (III)]. In the case of (II), the indole ring systems are nearly coplanar with the cyclo­penta­none rings [dihedral angles: = 0.58 (9) and 1.52 (8)°].

In all three compounds, as a result of the electron-withdrawing character of the phenyl­sulfonyl group, the N—C*sp* ^2^ bond lengths are longer than the mean value of 1.355 (14)Å for the N—C bond length (Allen *et al.*, 1987 ▸). Atom S1 has a distorted tetra­hedral configuration. The widening of the angle O1=S1=O2 and the narrowing of the angle N1—S1—C9 from ideal tetra­hedral values are attributed to the Thorpe–Ingold effect (Bassindale, 1984 ▸). The widening of the angles may be due to the repulsive inter­action between the two short S=O bonds.

In all three compounds, the expansion of the *ispo* angles at atoms C1, C3 and C4, and the contraction of the apical angles at atoms C2, C5 and C6 are caused by fusion of the smaller pyrrole ring with the six-membered benzene ring and the strain is taken up by the angular distortion rather than by bond-length distortion (Allen, 1981 ▸).

The sums of the bond angles around atoms N1 are 351.55 and 356.16° in (I), 359.86 and 359.29° in (II), and 352.79° in (III), indicating *sp* ^2^ hybridization. In all three compounds, the mol­ecular structure is stabilized by intra­molecular C—H⋯O hydrogen bonds which generate *S*(6) ring motifs with the sulfone O atom (Tables 1 ▸, 2 ▸ and 3 ▸). In addition to these, in compound (III), the mol­ecular structure is characterized by intra­molecular C25—Cl3⋯O2 halogen bonding (XB), between the solvent Cl atom (Cl3) and sulfone-group O atom (O2) [Cl3⋯O2 = 3.036 (2) Å and with a bond angle of 164.48 (14)°].

## Supra­molecular features   

**Figure 6:**
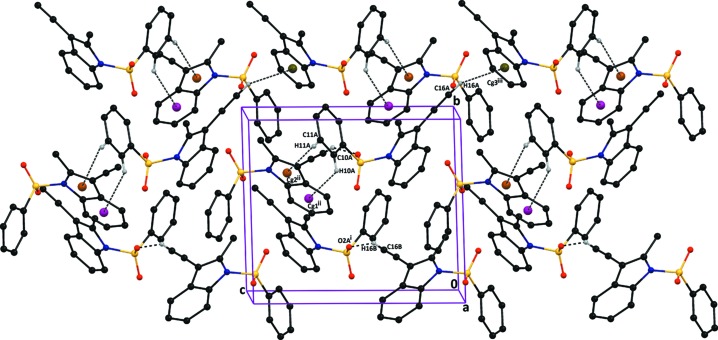
The crystal packing of compound (I), viewed down the *b* axis, showing C12*B*—H12*B*⋯O2*A* ^i^ inter­molecular hydrogen bond link the independent mol­ecules running parallel to the [101] direction and further inter­connected by C10*A*—H10*A*⋯*Cg*1^ii^, C11*A*—H11*A*⋯*Cg*2^ii^ and C16*A*—H16*A*⋯*Cg*3^iii^ inter­actions. *Cg*2 is the centre of the gravity of the pyrrole ring (atoms N1*B*/C1*B*/C6*B*/C7*B*/C8*B*), and *Cg*1 and *Cg*3 are the centres of the gravity of benzene rings C1*B*–C6*B* and C1*A*–C6*A*, respectively. [Symmetry codes: (i) −*x* + 1, *y* + 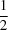, −*z* + 1; (ii) −*x* + 2, *y* − 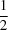, −*z* + 1; (iii) −*x* + 1, *y* + 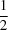, −*z*.]

In the crystal packing of compound (I), the mol­ecules are linked *via* inter­molecular C16*B*—H16*B*⋯O2*A*(−*x* + 1, *y* + 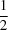, −*z* + 1) hydrogen bonds running parallel to the [101] direction. The crystal packing is further stabilized by inter­molecular C10*A*—H10*A*⋯*Cg*1, C11*A*—H11*A*⋯*Cg*2 and C16*A*—H16*A*⋯*Cg*3 inter­actions, with separations of 3.727 (2), 3.546 (2) and 3.699 (3) Å at (−*x* + 2, *y* − 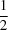, −*z* + 1) and (−*x* + 1, *y* + 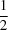, −*z*), respectively. *Cg*2 is the centre of gravity of pyrrole ring N1*B*/C1*B*/C6*B*/C7*B*/C8*B*, and *Cg*1 and *Cg*3 are the centres of gravity of benzene rings C1*B*–C6*B* and C1*A*–C6*A*, respectively. C—H⋯π inter­actions run parallel to the [210] direction (Table 1 ▸ and Fig. 6 ▸).

**Figure 7:**
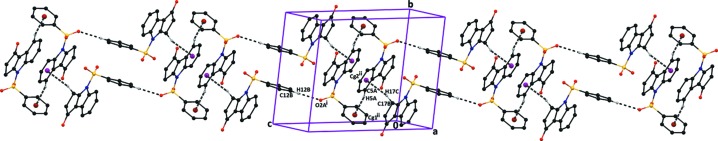
The crystal packing of compound (II), viewed down the *b* axis, showing C12*B*—H12*B*⋯O2*A* ^i^ inter­molecular hydrogen bond running parallel to the [101] direction and further inter­comnnected by C5*A*—H5*A*⋯*Cg*1^ii^ and C17*B*—H17*C*⋯*Cg*2^ii^ inter­actions. H atoms not involved in the hydrogen bonding have been omitted for clarity. *Cg*1 and *Cg*2 are the centres of the gravity of benzene rings C9*A*–C14*A* and C1*A*–C6*A*, respectively. [Symmetry codes: (i) *x* + 1, *y*, *z* − 1; (ii) −*x* + 1, −*y* + 1, −*z* + 1.]

In the crystal packing of compound (II), the independent mol­ecules (*A* and *B*) are linked by inter­molecular C12*B*—H12*B*⋯O2*A*(*x* + 1, *y*, *z* − 1) hydrogen bonds and are further connected by C5*A*—H5*A*⋯*Cg*1 and C17*B*—H17*C*⋯*Cg*2 inter­actions, with separations of 3.550 (2) and 3.729 (2) Å at (−*x* + 1, −*y* + 1, -*z+1) (*Cg**1 and *Cg*2 are the centres of gravity of benzene rings C9*A*–C14*A* and C1*A*–C6*A*), respectively). The C12*B*—H12*B*⋯O2*A* and C17*B*—H17*C*⋯*Cg*2 inter­actions run parallel to the [101] direction and C5*A*—H5*A*⋯*Cg*1 inter­actions run along the [0
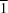
1] direction (Table 2 ▸ and Fig. 7 ▸), respectively.

**Figure 8:**
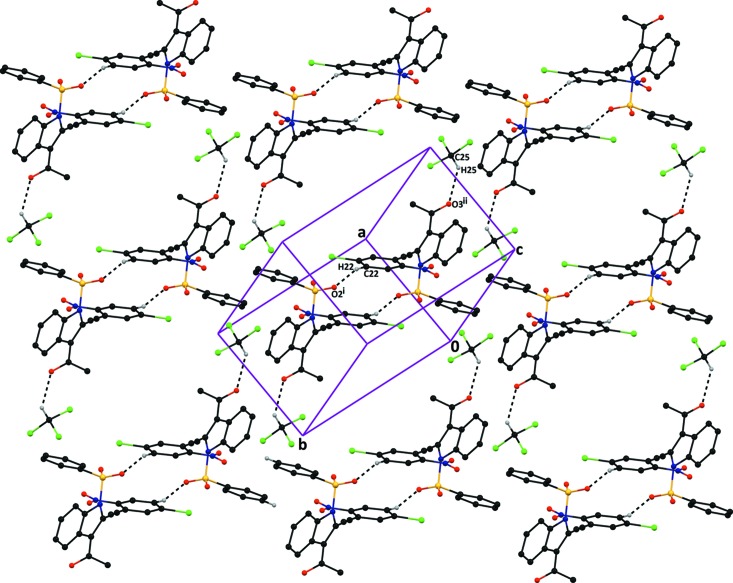
The crystal packing of compound (III), viewed down the *c* axis, showing C22—H22⋯O2^i^ inter­molecular hydrogen bonds, which results in 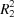
(22) inversion dimers forms a sheet lying parallel to the [1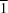 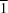] direction. In addition, the solvent mol­ecule inter­acts with the organic mol­ecule linked *via* a C25—H25⋯O3^ii^ hydrogen bond. H atoms not involved in the hydrogen bonding have been omitted for clarity. [Symmetry codes: (i) −*x* + 1, −*y* + 1, −*z* + 1; (ii) −*x* + 1, −*y* + 1, −*z*.]

In the crystal of compound (III), mol­ecules are linked *via* C22—H22⋯O2(−*x* + 1, −*y* + 1, −*z* + 1) inter­molecular hydrogen bonds which generates 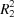
(22) inversion dimers. In addition, the chloro­form solvent mol­ecule is linked to the organic mol­ecule by a C25—H25⋯O3 hydrogen bond (Bernstein *et al.*, 1995 ▸) involving the O atom of the ethanone group (Table 3 ▸ and Fig. 8 ▸).

## Synthesis and crystallization   

### Compound (I)   

A solution of [(3-acetyl-1-phenyl­sulfanyl-1*H*-indol-2-yl)meth­yl]tri­phenyl­phospho­nium ylide (0.5 g, 9 mmol) in dry toluene (20 ml) was refluxed for 12 h under an N_2_ atmosphere. After consumption of the starting material [monitered by thin-layer chromatography (TLC)], removal of the solvent in *vacuo* followed by column chromatographic purification (silica gel, EtOAc–hexane 1:9 *v*/*v*) gave (I) (yield 1.30 g, 29%) as a colourless solid. Single crystals suitable for X-ray diffraction were prepared by slow evaporation of a solution of compound (I) in ethyl acetate at room temperature (m.p. 383–385 K).

### Compound (II)   

Reaction of 2-bromo­methyl-1-(1-phenyl­sulfonyl-1*H*-indol-3-yl)ethan-1-one (0.2 g, 5 mmol) with K_2_CO_3_ (0.35 g, 5 mmol) in aceto­nitrile was carried out under reflux for 8 h under an N_2_ atmosphere. After the consumption of the starting material (monitered by TLC), the reaction mass was poured over crushed ice and extracted with di­chloro­methane (2 × 15 ml). The organic layers were combined and washed with brine solution (2 × 20 ml) and dried (Na_2_SO_4_). The crude product was purified by column chromatography (silica gel, EtOAc–hexane 1:4 *v*/*v*) to give (II) (yield 1.40 g, 88%) as a white solid. Single crystals suitable for X-ray diffraction were prepared by slow evaporation of a solution of compound (II) in ethyl acetate at room temperature (m.p. 475–481 K).

### Compound (III)   

A solution of [(3-acetyl-1-phenyl­sufanyl-1*H*-indol-2-yl)meth­yl]tri­phenyl­phosphonium ylide (3 g, 5.23 mmol) and 5-chloro­nitro­benzaldehyde (1.06 g, 5.75 mmol) in dry chloro­form (50 ml) was refluxed for 10 h under an N_2_ atmosphere. Removal of the solvent in *vacuo* followed by titration of the crude product with methonal (10 ml), gave (III) (yield 2.29 g, 91%) as a yellow solid. Single crystals suitable for X-ray diffraction were prepared by slow evaporation of a solution of compound (III) in chloro­form at room temperature (m.p. 439–441 K).

## Refinement   

Crystal data, data collection and structure refinement details for compounds (I), (II) and (III) are summarized in Table 4 ▸. The positions of the H atoms were localized from the difference electron-density maps and their distances were geometrically constrained. H atoms bound to the C atoms were treated as riding atoms, with C—H = 0.93, 0.96, 0.97 and 0.98 Å for aryl, methyl, methyl­ene and methine H atoms, respectively, with *U*_iso_(H) = 1.5*U*_eq_(methyl C) and 1.2*U*_eq_(nonmethyl C). The rotation angles for methyl groups were optimized by least squares.

## Supplementary Material

Crystal structure: contains datablock(s) I, II, III, global. DOI: 10.1107/S2056989015014917/bg2558sup1.cif

Structure factors: contains datablock(s) I. DOI: 10.1107/S2056989015014917/bg2558Isup2.hkl

Structure factors: contains datablock(s) II. DOI: 10.1107/S2056989015014917/bg2558IIsup3.hkl

Structure factors: contains datablock(s) III. DOI: 10.1107/S2056989015014917/bg2558IIIsup4.hkl

Supporting information file. DOI: 10.1107/S2056989015014917/bg2558IIsup5.cml

Supporting information file. DOI: 10.1107/S2056989015014917/bg2558IIIsup6.cml

CCDC references: 1417660, 1417659, 1417658

Additional supporting information:  crystallographic information; 3D view; checkCIF report

## Figures and Tables

**Figure 2:**
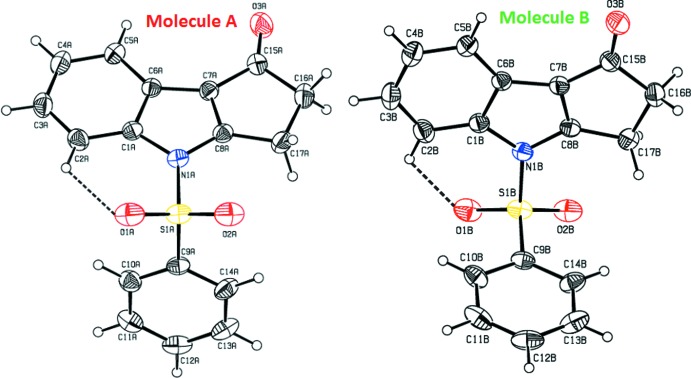
The mol­ecular structure of the compound (II), showing the atom-numbering scheme. The intra­molecular C2*A*—H2*A*⋯O2*A* and C2*B*—H2*B*⋯O2*B* inter­actions (mol­ecules *A* and *B*), which generate two *S*(6) ring motifs, are shown as dashed lines. Displacement ellipsoids are drawn at the 30% probability level.

**Figure 3:**
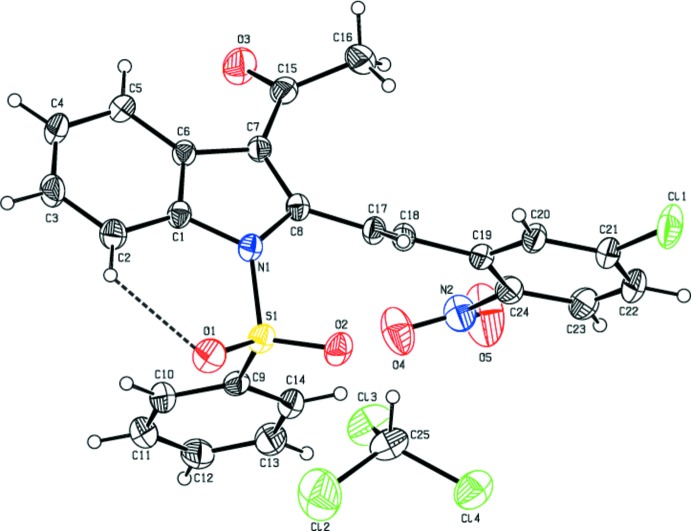
The mol­ecular structure of the compound (III), showing the atom-numbering scheme. The intra­molecular C2—H2⋯O2 inter­action, which generates an *S*(6) ring motif, is shown as a dashed line. Displacement ellipsoids are drawn at the 30% probability level.

**Figure 5:**
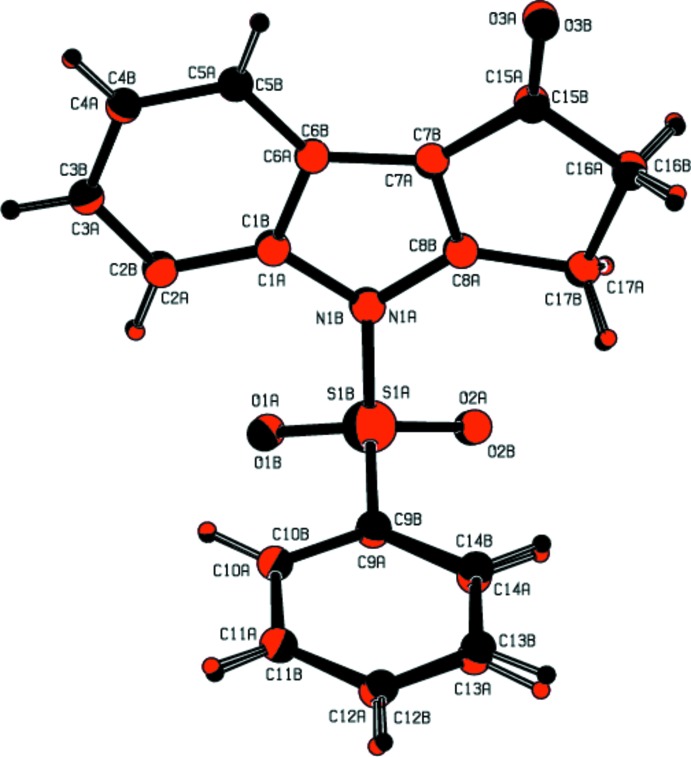
The mol­ecule *A* (red) and mol­ecule *B* (black) of title compound (II) overlapping with each other. H atoms are shown as spheres of arbitrary radius.

**Table 1:** Hydrogen-bond geometry (Å, °) for (I) *Cg*2 is the centroid of the pyrrole ring N1*A*/C1*A*/C6*A*/C7*A*/C8*A*, *Cg*1 and *Cg*3 are the centroids of the benzene rings C1*B*–C6*B* and C1*A*–C6*A*.

*D*—H⋯*A*	*D*—H	H⋯*A*	*D*⋯*A*	*D*—H⋯*A*
C2*A*—H2*A*⋯O1*A*	0.93	2.36	2.941 (3)	121
C2*B*—H2*B*⋯O1*B*	0.93	2.38	2.957 (3)	120
C16*B*—H16*B*⋯O2*A* ^i^	0.93	2.43	3.334 (3)	153
C10*A*—H10*A*⋯*Cg*1^ii^	0.93	2.95	3.728 (2)	142
C11*A*—H11*A*⋯*Cg*2^ii^	0.93	2.74	3.546 (2)	145
C16*A*—H16*A*⋯*Cg*3^iii^	0.93	2.88	3.699 (3)	148

Symmetry codes: (i) 
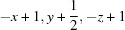
; (ii) 
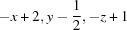
; (iii) 
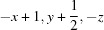
.

**Table 2:** Hydrogen-bond geometry (Å, °) for (II) *Cg*1 and *Cg*2 are the centroids of the benzene rings C9*A*–C14*A* and C1*A*–C6*A*.

*D*—H⋯*A*	*D*—H	H⋯*A*	*D*⋯*A*	*D*—H⋯*A*
C2*A*—H2*A*⋯O1*A*	0.93	2.44	3.007 (2)	119
C2*B*—H2*B*⋯O1*B*	0.93	2.44	3.010 (2)	120
C12*B*—H12*B*⋯O2*A* ^i^	0.93	2.46	3.369 (3)	166
C5*A*—H5*A*⋯*Cg*1^ii^	0.93	2.65	3.550 (2)	164
C17*B*—H17*C*⋯*Cg*2^ii^	0.97	2.85	3.729 (2)	151

Symmetry codes: (i) 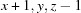; (ii) 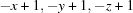.

**Table 3:** Hydrogen-bond geometry (Å, °) for (III)

*D*—H⋯*A*	*D*—H	H⋯*A*	*D*⋯*A*	*D*—H⋯*A*
C2—H2⋯O1	0.93	2.32	2.903 (4)	121
C22—H22⋯O2^i^	0.93	2.51	3.412 (4)	162
C25—H25⋯O3^ii^	0.98	2.49	3.283 (4)	138

Symmetry codes: (i) 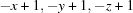; (ii) 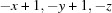.

**Table 4:** Experimental details

	(I)	(II)	(III)
Crystal data			
Chemical formula	C_17_H_13_NO_2_S	C_17_H_13_NO_3_S	C_24_H_17_ClN_2_O_5_S·CHCl_3_
*M*_r_	295.34	311.34	600.27
Crystal system, space group	Monoclinic, *P*2_1_	Triclinic, *P* 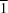	Triclinic, *P* 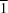
Temperature (K)	296	296	296
*a*, *b*, *c* (Å)	12.1786 (5), 10.2422 (5), 12.6306 (5)	9.8708 (6), 12.3914 (7), 13.1457 (12)	9.5856 (3), 11.2767 (4), 13.1782 (4)
α, β, γ (°)	90, 113.082 (2), 90	102.706 (3), 96.552 (3), 111.989 (2)	104.9070 (11), 108.2350 (9), 91.581 (1)
*V* (Å^3^)	1449.36 (11)	1419.70 (18)	1298.31 (7)
*Z*	4	4	2
Radiation type	Mo *K*α	Mo *K*α	Mo *K*α
μ (mm^−1^)	0.23	0.24	0.58
Crystal size (mm)	0.35 × 0.30 × 0.25	0.35 × 0.30 × 0.25	0.35 × 0.30 × 0.25

Data collection			
Diffractometer	Bruker Kappa APEXII CCD diffractometer	Bruker Kappa APEXII CCD diffractometer	Bruker Kappa APEXII CCD diffractometer
Absorption correction	Multi-scan (*SADABS*; Bruker, 2008 ▸)	Multi-scan (*SADABS*; Bruker, 2008 ▸)	Multi-scan (*SADABS*; Bruker, 2008 ▸)
*T*_min_, *T*_max_	0.924, 0.945	0.919, 0.942	0.817, 0.866
No. of measured, independent and observed [*I* > 2σ(*I*)] reflections	12944, 5750, 5372	20747, 5869, 4993	25757, 4579, 4054
*R*_int_	0.024	0.028	0.019
(sin θ/λ)_max_ (Å^−1^)	0.639	0.628	0.595

Refinement			
*R*[*F* ^2^ > 2σ(*F* ^2^)], *wR*(*F* ^2^), *S*	0.029, 0.080, 1.02	0.038, 0.105, 1.04	0.049, 0.136, 1.05
No. of reflections	5750	5869	4579
No. of parameters	389	397	335
No. of restraints	1	0	0
H-atom treatment	H-atom parameters constrained	H-atom parameters constrained	H-atom parameters constrained
Δρ_max_, Δρ_min_ (e Å^−3^)	0.16, −0.25	0.22, −0.46	0.99, −0.77
Absolute structure	Flack (1983 ▸), 2406 Friedel pairs	–	–
Absolute structure parameter	0.01 (4)	–	–

Computer programs: *APEX2* (Bruker, 2008 ▸), *SAINT* (Bruker, 2008 ▸), *SHELXS97* (Sheldrick, 2008 ▸), *ORTEP-3 for Windows* (Farrugia, 2012 ▸) and *Mercury* (Macrae *et al.*, 2008 ▸), *SHELXL97* (Sheldrick, 2008 ▸) and *PLATON* (Spek, 2009 ▸).

## supplementary crystallographic information

### (I) 3-Ethnyl-2-methyl-1-phenylsulfonyl-1H-indole Crystal data

**Table:**

C_17_H_13_NO_2_S	*F*(000) = 616
*M**_r_* = 295.34	*D*_x_ = 1.354 Mg m^−^^3^
Monoclinic, *P*2_1_	Mo *K*α radiation, λ = 0.71073 Å
Hall symbol: P 2yb	Cell parameters from 5750 reflections
*a* = 12.1786 (5) Å	θ = 1.8–27.0°
*b* = 10.2422 (5) Å	µ = 0.23 mm^−^^1^
*c* = 12.6306 (5) Å	*T* = 296 K
β = 113.082 (2)°	Block, colourless
*V* = 1449.36 (11) Å^3^	0.35 × 0.30 × 0.25 mm
*Z* = 4	

### (I) 3-Ethnyl-2-methyl-1-phenylsulfonyl-1H-indole Data collection

**Table:**

Bruker Kappa APEXII CCD diffractometer	5750 independent reflections
Radiation source: fine-focus sealed tube	5372 reflections with *I* > 2σ(*I*)
Graphite monochromator	*R*_int_ = 0.024
ω & φ scans	θ_max_ = 27.0°, θ_min_ = 1.8°
Absorption correction: multi-scan (SADABS; Bruker, 2008)	*h* = −15→15
*T*_min_ = 0.924, *T*_max_ = 0.945	*k* = −13→11
12944 measured reflections	*l* = −16→13

### (I) 3-Ethnyl-2-methyl-1-phenylsulfonyl-1H-indole Refinement

**Table:**

Refinement on *F*^2^	Secondary atom site location: difference Fourier map
Least-squares matrix: full	Hydrogen site location: inferred from neighbouring sites
*R*[*F*^2^ > 2σ(*F*^2^)] = 0.029	H-atom parameters constrained
*wR*(*F*^2^) = 0.080	*w* = 1/[σ^2^(*F*_o_^2^) + (0.0507*P*)^2^ + 0.0899*P*] where *P* = (*F*_o_^2^ + 2*F*_c_^2^)/3
*S* = 1.02	(Δ/σ)_max_ = 0.001
5750 reflections	Δρ_max_ = 0.16 e Å^−^^3^
389 parameters	Δρ_min_ = −0.25 e Å^−^^3^
1 restraint	Absolute structure: Flack (1983), ???? Friedel pairs
Primary atom site location: structure-invariant direct methods	Absolute structure parameter: 0.01 (4)

### (I) 3-Ethnyl-2-methyl-1-phenylsulfonyl-1H-indole Special details

**Table:**

Geometry. All e.s.d.'s (except the e.s.d. in the dihedral angle between two l.s. planes) are estimated using the full covariance matrix. The cell e.s.d.'s are taken into account individually in the estimation of e.s.d.'s in distances, angles and torsion angles; correlations between e.s.d.'s in cell parameters are only used when they are defined by crystal symmetry. An approximate (isotropic) treatment of cell e.s.d.'s is used for estimating e.s.d.'s involving l.s. planes.
Refinement. Refinement of *F*^2^ against ALL reflections. The weighted *R*-factor *wR* and goodness of fit *S* are based on *F*^2^, conventional *R*-factors *R* are based on *F*, with *F* set to zero for negative *F*^2^. The threshold expression of *F*^2^ > σ(*F*^2^) is used only for calculating *R*-factors(gt) *etc*. and is not relevant to the choice of reflections for refinement. *R*-factors based on *F*^2^ are statistically about twice as large as those based on *F*, and *R*- factors based on ALL data will be even larger.

### (I) 3-Ethnyl-2-methyl-1-phenylsulfonyl-1H-indole Fractional atomic coordinates and isotropic or equivalent isotropic displacement parameters (Å^2^)

**Table:**

	*x*	*y*	*z*	*U*_iso_*/*U*_eq_	
C1A	0.65808 (13)	0.73762 (18)	0.29191 (13)	0.0410 (3)	
C1B	0.95152 (14)	1.07543 (17)	0.20687 (14)	0.0455 (4)	
C2A	0.73969 (17)	0.6367 (2)	0.32455 (17)	0.0550 (4)	
H2A	0.7426	0.5771	0.3811	0.066*	
C2B	1.04368 (16)	0.9877 (2)	0.22107 (18)	0.0596 (5)	
H2B	1.0758	0.9783	0.1657	0.072*	
C3A	0.81721 (17)	0.6290 (2)	0.26835 (19)	0.0658 (5)	
H3A	0.8732	0.5620	0.2878	0.079*	
C3B	1.0845 (2)	0.9162 (2)	0.3204 (2)	0.0747 (7)	
H3B	1.1461	0.8569	0.3323	0.090*	
C4A	0.81412 (17)	0.7173 (2)	0.18443 (18)	0.0621 (5)	
H4A	0.8679	0.7091	0.1491	0.075*	
C4B	1.0382 (2)	0.9282 (3)	0.4041 (2)	0.0775 (7)	
H4B	1.0688	0.8775	0.4704	0.093*	
C5A	0.73243 (16)	0.8168 (2)	0.15291 (15)	0.0521 (4)	
H5A	0.7300	0.8761	0.0963	0.062*	
C5B	0.94725 (19)	1.0145 (2)	0.38970 (16)	0.0636 (5)	
H5B	0.9154	1.0227	0.4453	0.076*	
C6A	0.65311 (13)	0.82726 (17)	0.20735 (13)	0.0404 (3)	
C6B	0.90403 (15)	1.08907 (18)	0.29039 (14)	0.0477 (4)	
C7A	0.55590 (14)	0.91571 (16)	0.19224 (13)	0.0431 (4)	
C7B	0.81248 (14)	1.18826 (17)	0.25068 (14)	0.0472 (4)	
C8A	0.50461 (14)	0.88117 (17)	0.26651 (13)	0.0431 (4)	
C8B	0.80488 (14)	1.23229 (19)	0.14747 (14)	0.0482 (4)	
C9A	0.69568 (14)	0.84083 (17)	0.54842 (13)	0.0422 (4)	
C9B	0.78684 (16)	1.02723 (18)	−0.08004 (14)	0.0453 (4)	
C10A	0.81214 (16)	0.7967 (2)	0.59661 (16)	0.0546 (4)	
H10A	0.8308	0.7109	0.5861	0.066*	
C10B	0.82856 (17)	0.90137 (19)	−0.07879 (16)	0.0531 (4)	
H10B	0.9097	0.8830	−0.0436	0.064*	
C11A	0.89979 (17)	0.8829 (2)	0.66059 (18)	0.0633 (5)	
H11A	0.9787	0.8550	0.6941	0.076*	
C11B	0.7474 (2)	0.8039 (2)	−0.13078 (18)	0.0630 (5)	
H11B	0.7738	0.7186	−0.1305	0.076*	
C12A	0.87192 (17)	1.0095 (2)	0.67542 (17)	0.0602 (5)	
H12A	0.9320	1.0669	0.7186	0.072*	
C12B	0.62746 (19)	0.8317 (2)	−0.18316 (17)	0.0641 (5)	
H12B	0.5734	0.7652	−0.2184	0.077*	
C13A	0.75599 (17)	1.0519 (2)	0.62696 (18)	0.0593 (5)	
H13A	0.7378	1.1379	0.6375	0.071*	
C13B	0.58749 (19)	0.9568 (2)	−0.18365 (19)	0.0661 (5)	
H13B	0.5063	0.9747	−0.2191	0.079*	
C14A	0.66630 (15)	0.9678 (2)	0.56280 (17)	0.0538 (4)	
H14A	0.5875	0.9961	0.5298	0.065*	
C14B	0.66648 (18)	1.0563 (2)	−0.13216 (16)	0.0574 (5)	
H14B	0.6395	1.1413	−0.1324	0.069*	
C15A	0.51574 (16)	1.0165 (2)	0.10820 (16)	0.0534 (4)	
C15B	0.74452 (15)	1.2338 (2)	0.31326 (16)	0.0584 (5)	
C16A	0.4818 (2)	1.0963 (3)	0.0369 (2)	0.0744 (6)	
H16A	0.452	1.157	−0.022	0.088 (10)*	
C16B	0.6914 (2)	1.2689 (3)	0.3686 (2)	0.0764 (7)	
H16B	0.647	1.302	0.41	0.090*	
C17A	0.39623 (17)	0.9400 (2)	0.27481 (18)	0.0612 (5)	
H17C	0.3627	1.0036	0.2149	0.092*	
H17D	0.4178	0.9815	0.3484	0.092*	
H17E	0.3385	0.8728	0.2665	0.092*	
C17B	0.7258 (2)	1.3385 (2)	0.07802 (18)	0.0715 (6)	
H17F	0.6788	1.3719	0.1177	0.107*	
H17G	0.6739	1.3046	0.0045	0.107*	
H17H	0.7736	1.4075	0.0670	0.107*	
N1A	0.56467 (11)	0.76926 (13)	0.32876 (11)	0.0424 (3)	
N1B	0.89185 (12)	1.16606 (15)	0.11823 (11)	0.0474 (3)	
O1A	0.62039 (14)	0.60415 (13)	0.48518 (13)	0.0635 (4)	
O1B	1.00485 (13)	1.11237 (17)	−0.00198 (14)	0.0705 (4)	
O2A	0.47181 (11)	0.77339 (15)	0.47241 (12)	0.0618 (4)	
O2B	0.84226 (15)	1.27284 (14)	−0.07179 (13)	0.0726 (4)	
S1A	0.58058 (4)	0.73619 (4)	0.46403 (4)	0.04641 (11)	
S1B	0.88932 (4)	1.15298 (5)	−0.01502 (4)	0.05211 (12)	

### (I) 3-Ethnyl-2-methyl-1-phenylsulfonyl-1H-indole Atomic displacement parameters (Å^2^)

**Table:**

	*U*^11^	*U*^22^	*U*^33^	*U*^12^	*U*^13^	*U*^23^
C1A	0.0404 (7)	0.0413 (8)	0.0444 (7)	−0.0043 (7)	0.0201 (6)	−0.0060 (7)
C1B	0.0408 (8)	0.0444 (9)	0.0476 (8)	−0.0031 (7)	0.0135 (6)	−0.0041 (7)
C2A	0.0577 (10)	0.0495 (11)	0.0631 (10)	0.0076 (8)	0.0292 (8)	0.0053 (8)
C2B	0.0516 (10)	0.0534 (12)	0.0727 (12)	0.0080 (8)	0.0231 (9)	−0.0050 (9)
C3A	0.0541 (10)	0.0669 (14)	0.0827 (13)	0.0152 (9)	0.0334 (10)	−0.0005 (11)
C3B	0.0623 (12)	0.0602 (14)	0.0885 (15)	0.0208 (10)	0.0154 (11)	0.0052 (11)
C4A	0.0505 (9)	0.0787 (14)	0.0676 (11)	0.0004 (10)	0.0343 (8)	−0.0073 (11)
C4B	0.0792 (14)	0.0719 (15)	0.0673 (13)	0.0160 (12)	0.0134 (11)	0.0193 (11)
C5A	0.0503 (9)	0.0623 (11)	0.0501 (9)	−0.0100 (8)	0.0267 (7)	−0.0039 (8)
C5B	0.0689 (12)	0.0687 (13)	0.0496 (10)	0.0017 (10)	0.0194 (9)	0.0073 (9)
C6A	0.0396 (7)	0.0420 (9)	0.0384 (7)	−0.0082 (6)	0.0141 (6)	−0.0065 (6)
C6B	0.0440 (8)	0.0470 (10)	0.0471 (8)	−0.0034 (7)	0.0124 (7)	−0.0046 (7)
C7A	0.0433 (8)	0.0418 (9)	0.0409 (8)	−0.0052 (6)	0.0131 (6)	−0.0044 (6)
C7B	0.0407 (8)	0.0506 (10)	0.0462 (8)	−0.0018 (7)	0.0125 (6)	−0.0086 (7)
C8A	0.0425 (8)	0.0419 (9)	0.0427 (8)	−0.0017 (7)	0.0144 (6)	−0.0065 (7)
C8B	0.0455 (8)	0.0455 (9)	0.0473 (8)	0.0012 (8)	0.0113 (6)	−0.0081 (8)
C9A	0.0443 (8)	0.0480 (10)	0.0393 (7)	−0.0008 (7)	0.0218 (6)	0.0026 (7)
C9B	0.0525 (9)	0.0507 (10)	0.0378 (7)	−0.0072 (7)	0.0230 (7)	−0.0006 (7)
C10A	0.0515 (9)	0.0533 (11)	0.0568 (9)	0.0074 (8)	0.0188 (8)	0.0037 (8)
C10B	0.0536 (9)	0.0554 (11)	0.0522 (9)	0.0028 (8)	0.0227 (8)	−0.0013 (8)
C11A	0.0434 (9)	0.0752 (14)	0.0626 (11)	0.0096 (9)	0.0112 (8)	0.0007 (10)
C11B	0.0751 (12)	0.0493 (11)	0.0639 (11)	−0.0008 (9)	0.0266 (10)	−0.0059 (9)
C12A	0.0498 (10)	0.0721 (14)	0.0567 (10)	−0.0108 (9)	0.0189 (8)	−0.0092 (10)
C12B	0.0693 (12)	0.0581 (13)	0.0582 (11)	−0.0147 (10)	0.0179 (9)	−0.0066 (9)
C13A	0.0557 (10)	0.0513 (11)	0.0694 (12)	0.0002 (8)	0.0229 (9)	−0.0096 (9)
C13B	0.0549 (10)	0.0681 (13)	0.0650 (12)	−0.0026 (10)	0.0124 (9)	−0.0040 (10)
C14A	0.0434 (8)	0.0537 (11)	0.0633 (11)	0.0051 (8)	0.0199 (8)	−0.0035 (9)
C14B	0.0601 (10)	0.0552 (11)	0.0541 (10)	0.0034 (9)	0.0195 (8)	−0.0011 (9)
C15A	0.0538 (10)	0.0519 (11)	0.0516 (9)	0.0011 (8)	0.0175 (8)	−0.0012 (8)
C15B	0.0469 (8)	0.0737 (13)	0.0514 (9)	0.0012 (10)	0.0158 (7)	−0.0106 (10)
C16A	0.0803 (14)	0.0700 (15)	0.0687 (13)	0.0148 (12)	0.0247 (11)	0.0235 (12)
C16B	0.0602 (11)	0.108 (2)	0.0633 (11)	0.0105 (12)	0.0270 (10)	−0.0110 (12)
C17A	0.0548 (10)	0.0672 (13)	0.0642 (11)	0.0137 (9)	0.0260 (9)	−0.0020 (10)
C17B	0.0828 (14)	0.0601 (14)	0.0607 (12)	0.0248 (11)	0.0164 (10)	0.0010 (10)
N1A	0.0429 (7)	0.0427 (8)	0.0470 (7)	−0.0017 (5)	0.0235 (6)	−0.0026 (6)
N1B	0.0501 (7)	0.0456 (8)	0.0453 (7)	−0.0009 (6)	0.0175 (6)	−0.0042 (6)
O1A	0.0804 (9)	0.0448 (8)	0.0740 (8)	−0.0075 (6)	0.0398 (7)	0.0119 (6)
O1B	0.0614 (7)	0.0857 (11)	0.0780 (9)	−0.0214 (8)	0.0418 (7)	−0.0109 (8)
O2A	0.0527 (7)	0.0776 (10)	0.0691 (8)	−0.0135 (6)	0.0389 (6)	−0.0025 (7)
O2B	0.0989 (11)	0.0560 (9)	0.0652 (8)	−0.0141 (7)	0.0348 (8)	0.0135 (7)
S1A	0.0495 (2)	0.0472 (2)	0.0508 (2)	−0.00875 (18)	0.02854 (17)	0.00263 (19)
S1B	0.0593 (2)	0.0526 (3)	0.0507 (2)	−0.0126 (2)	0.02839 (19)	0.00082 (19)

### (I) 3-Ethnyl-2-methyl-1-phenylsulfonyl-1H-indole Geometric parameters (Å, º)

**Table:**

C1A—C2A	1.380 (2)	C9B—C10B	1.383 (3)
C1A—C6A	1.392 (2)	C9B—S1B	1.7570 (18)
C1A—N1A	1.425 (2)	C10A—C11A	1.377 (3)
C1B—C2B	1.393 (2)	C10A—H10A	0.9300
C1B—C6B	1.395 (3)	C10B—C11B	1.377 (3)
C1B—N1B	1.416 (2)	C10B—H10B	0.9300
C2A—C3A	1.388 (3)	C11A—C12A	1.372 (3)
C2A—H2A	0.9300	C11A—H11A	0.9300
C2B—C3B	1.367 (3)	C11B—C12B	1.376 (3)
C2B—H2B	0.9300	C11B—H11B	0.9300
C3A—C4A	1.383 (3)	C12A—C13A	1.371 (3)
C3A—H3A	0.9300	C12A—H12A	0.9300
C3B—C4B	1.386 (4)	C12B—C13B	1.370 (3)
C3B—H3B	0.9300	C12B—H12B	0.9300
C4A—C5A	1.369 (3)	C13A—C14A	1.377 (3)
C4A—H4A	0.9300	C13A—H13A	0.9300
C4B—C5B	1.372 (3)	C13B—C14B	1.377 (3)
C4B—H4B	0.9300	C13B—H13B	0.9300
C5A—C6A	1.392 (2)	C14A—H14A	0.9300
C5A—H5A	0.9300	C14B—H14B	0.9300
C5B—C6B	1.384 (3)	C15A—C16A	1.166 (3)
C5B—H5B	0.9300	C15B—C16B	1.179 (3)
C6A—C7A	1.443 (2)	C16A—H16A	0.9300
C6B—C7B	1.445 (2)	C16B—H16B	0.9300
C7A—C8A	1.362 (2)	C17A—H17C	0.9600
C7A—C15A	1.423 (2)	C17A—H17D	0.9600
C7B—C8B	1.348 (2)	C17A—H17E	0.9600
C7B—C15B	1.428 (3)	C17B—H17F	0.9600
C8A—N1A	1.420 (2)	C17B—H17G	0.9600
C8A—C17A	1.492 (2)	C17B—H17H	0.9600
C8B—N1B	1.423 (2)	N1A—S1A	1.6769 (14)
C8B—C17B	1.490 (3)	N1B—S1B	1.6764 (14)
C9A—C14A	1.380 (3)	O1A—S1A	1.4260 (15)
C9A—C10A	1.382 (2)	O1B—S1B	1.4136 (16)
C9A—S1A	1.7545 (17)	O2A—S1A	1.4217 (14)
C9B—C14B	1.383 (3)	O2B—S1B	1.4239 (15)
			
C2A—C1A—C6A	122.21 (15)	C9B—C10B—H10B	120.7
C2A—C1A—N1A	130.26 (16)	C12A—C11A—C10A	120.66 (18)
C6A—C1A—N1A	107.49 (14)	C12A—C11A—H11A	119.7
C2B—C1B—C6B	121.28 (17)	C10A—C11A—H11A	119.7
C2B—C1B—N1B	131.47 (18)	C12B—C11B—C10B	120.5 (2)
C6B—C1B—N1B	107.21 (14)	C12B—C11B—H11B	119.8
C1A—C2A—C3A	116.37 (18)	C10B—C11B—H11B	119.8
C1A—C2A—H2A	121.8	C11A—C12A—C13A	120.34 (19)
C3A—C2A—H2A	121.8	C11A—C12A—H12A	119.8
C3B—C2B—C1B	116.7 (2)	C13A—C12A—H12A	119.8
C3B—C2B—H2B	121.7	C13B—C12B—C11B	120.26 (19)
C1B—C2B—H2B	121.7	C13B—C12B—H12B	119.9
C4A—C3A—C2A	122.36 (19)	C11B—C12B—H12B	119.9
C4A—C3A—H3A	118.8	C12A—C13A—C14A	120.34 (19)
C2A—C3A—H3A	118.8	C12A—C13A—H13A	119.8
C2B—C3B—C4B	122.8 (2)	C14A—C13A—H13A	119.8
C2B—C3B—H3B	118.6	C12B—C13B—C14B	120.60 (19)
C4B—C3B—H3B	118.6	C12B—C13B—H13B	119.7
C5A—C4A—C3A	120.52 (18)	C14B—C13B—H13B	119.7
C5A—C4A—H4A	119.7	C13A—C14A—C9A	118.69 (16)
C3A—C4A—H4A	119.7	C13A—C14A—H14A	120.7
C5B—C4B—C3B	120.3 (2)	C9A—C14A—H14A	120.7
C5B—C4B—H4B	119.8	C13B—C14B—C9B	118.61 (19)
C3B—C4B—H4B	119.8	C13B—C14B—H14B	120.7
C4A—C5A—C6A	118.65 (18)	C9B—C14B—H14B	120.7
C4A—C5A—H5A	120.7	C16A—C15A—C7A	178.1 (2)
C6A—C5A—H5A	120.7	C16B—C15B—C7B	177.4 (2)
C4B—C5B—C6B	118.5 (2)	C15A—C16A—H16A	177
C4B—C5B—H5B	120.8	C15B—C16B—H16B	177.4
C6B—C5B—H5B	120.8	C8A—C17A—H17C	109.5
C1A—C6A—C5A	119.88 (16)	C8A—C17A—H17D	109.5
C1A—C6A—C7A	107.60 (15)	H17C—C17A—H17D	109.5
C5A—C6A—C7A	132.49 (16)	C8A—C17A—H17E	109.5
C5B—C6B—C1B	120.46 (18)	H17C—C17A—H17E	109.5
C5B—C6B—C7B	132.06 (18)	H17D—C17A—H17E	109.5
C1B—C6B—C7B	107.47 (15)	C8B—C17B—H17F	109.5
C8A—C7A—C15A	125.88 (17)	C8B—C17B—H17G	109.5
C8A—C7A—C6A	108.66 (15)	H17F—C17B—H17G	109.5
C15A—C7A—C6A	125.34 (17)	C8B—C17B—H17H	109.5
C8B—C7B—C15B	126.23 (17)	H17F—C17B—H17H	109.5
C8B—C7B—C6B	108.78 (15)	H17G—C17B—H17H	109.5
C15B—C7B—C6B	124.97 (17)	C8A—N1A—C1A	107.85 (13)
C7A—C8A—N1A	108.38 (14)	C8A—N1A—S1A	123.78 (11)
C7A—C8A—C17A	126.94 (17)	C1A—N1A—S1A	119.91 (11)
N1A—C8A—C17A	124.41 (16)	C1B—N1B—C8B	108.10 (14)
C7B—C8B—N1B	108.40 (15)	C1B—N1B—S1B	122.90 (12)
C7B—C8B—C17B	126.67 (17)	C8B—N1B—S1B	125.17 (11)
N1B—C8B—C17B	124.81 (17)	O2A—S1A—O1A	119.90 (9)
C14A—C9A—C10A	121.65 (17)	O2A—S1A—N1A	106.43 (8)
C14A—C9A—S1A	117.98 (13)	O1A—S1A—N1A	106.11 (8)
C10A—C9A—S1A	120.35 (14)	O2A—S1A—C9A	109.69 (8)
C14B—C9B—C10B	121.46 (17)	O1A—S1A—C9A	109.48 (9)
C14B—C9B—S1B	119.38 (15)	N1A—S1A—C9A	103.96 (7)
C10B—C9B—S1B	119.16 (14)	O1B—S1B—O2B	119.87 (10)
C11A—C10A—C9A	118.32 (18)	O1B—S1B—N1B	106.31 (8)
C11A—C10A—H10A	120.8	O2B—S1B—N1B	106.38 (9)
C9A—C10A—H10A	120.8	O1B—S1B—C9B	109.32 (9)
C11B—C10B—C9B	118.58 (18)	O2B—S1B—C9B	108.95 (9)
C11B—C10B—H10B	120.7	N1B—S1B—C9B	104.95 (8)
			
C6A—C1A—C2A—C3A	0.0 (3)	C11B—C12B—C13B—C14B	0.2 (3)
N1A—C1A—C2A—C3A	177.20 (17)	C12A—C13A—C14A—C9A	−0.1 (3)
C6B—C1B—C2B—C3B	0.1 (3)	C10A—C9A—C14A—C13A	0.0 (3)
N1B—C1B—C2B—C3B	177.65 (19)	S1A—C9A—C14A—C13A	−178.76 (15)
C1A—C2A—C3A—C4A	0.3 (3)	C12B—C13B—C14B—C9B	0.0 (3)
C1B—C2B—C3B—C4B	0.1 (3)	C10B—C9B—C14B—C13B	0.0 (3)
C2A—C3A—C4A—C5A	−0.4 (3)	S1B—C9B—C14B—C13B	179.41 (16)
C2B—C3B—C4B—C5B	0.0 (4)	C7A—C8A—N1A—C1A	1.52 (16)
C3A—C4A—C5A—C6A	0.2 (3)	C17A—C8A—N1A—C1A	175.90 (16)
C3B—C4B—C5B—C6B	−0.4 (4)	C7A—C8A—N1A—S1A	149.26 (12)
C2A—C1A—C6A—C5A	−0.1 (2)	C17A—C8A—N1A—S1A	−36.4 (2)
N1A—C1A—C6A—C5A	−177.89 (14)	C2A—C1A—N1A—C8A	−178.62 (17)
C2A—C1A—C6A—C7A	178.06 (15)	C6A—C1A—N1A—C8A	−1.11 (16)
N1A—C1A—C6A—C7A	0.31 (17)	C2A—C1A—N1A—S1A	32.2 (2)
C4A—C5A—C6A—C1A	0.0 (2)	C6A—C1A—N1A—S1A	−150.32 (11)
C4A—C5A—C6A—C7A	−177.66 (17)	C2B—C1B—N1B—C8B	−179.51 (18)
C4B—C5B—C6B—C1B	0.6 (3)	C6B—C1B—N1B—C8B	−1.71 (18)
C4B—C5B—C6B—C7B	−178.8 (2)	C2B—C1B—N1B—S1B	21.6 (3)
C2B—C1B—C6B—C5B	−0.4 (3)	C6B—C1B—N1B—S1B	−160.64 (12)
N1B—C1B—C6B—C5B	−178.51 (16)	C7B—C8B—N1B—C1B	1.83 (19)
C2B—C1B—C6B—C7B	179.05 (16)	C17B—C8B—N1B—C1B	177.97 (17)
N1B—C1B—C6B—C7B	0.97 (18)	C7B—C8B—N1B—S1B	160.16 (12)
C1A—C6A—C7A—C8A	0.64 (17)	C17B—C8B—N1B—S1B	−23.7 (3)
C5A—C6A—C7A—C8A	178.52 (16)	C8A—N1A—S1A—O2A	38.37 (14)
C1A—C6A—C7A—C15A	−175.48 (15)	C1A—N1A—S1A—O2A	−177.52 (13)
C5A—C6A—C7A—C15A	2.4 (3)	C8A—N1A—S1A—O1A	167.13 (12)
C5B—C6B—C7B—C8B	179.6 (2)	C1A—N1A—S1A—O1A	−48.76 (14)
C1B—C6B—C7B—C8B	0.16 (19)	C8A—N1A—S1A—C9A	−77.43 (13)
C5B—C6B—C7B—C15B	1.3 (3)	C1A—N1A—S1A—C9A	66.68 (14)
C1B—C6B—C7B—C15B	−178.11 (17)	C14A—C9A—S1A—O2A	−30.31 (17)
C15A—C7A—C8A—N1A	174.76 (15)	C10A—C9A—S1A—O2A	150.92 (15)
C6A—C7A—C8A—N1A	−1.33 (17)	C14A—C9A—S1A—O1A	−163.80 (14)
C15A—C7A—C8A—C17A	0.6 (3)	C10A—C9A—S1A—O1A	17.44 (17)
C6A—C7A—C8A—C17A	−175.53 (16)	C14A—C9A—S1A—N1A	83.17 (15)
C15B—C7B—C8B—N1B	177.02 (17)	C10A—C9A—S1A—N1A	−95.59 (15)
C6B—C7B—C8B—N1B	−1.22 (19)	C1B—N1B—S1B—O1B	−40.11 (15)
C15B—C7B—C8B—C17B	1.0 (3)	C8B—N1B—S1B—O1B	164.60 (16)
C6B—C7B—C8B—C17B	−177.26 (18)	C1B—N1B—S1B—O2B	−168.94 (13)
C14A—C9A—C10A—C11A	0.2 (3)	C8B—N1B—S1B—O2B	35.78 (17)
S1A—C9A—C10A—C11A	178.93 (15)	C1B—N1B—S1B—C9B	75.67 (14)
C14B—C9B—C10B—C11B	−0.1 (3)	C8B—N1B—S1B—C9B	−79.62 (17)
S1B—C9B—C10B—C11B	−179.54 (15)	C14B—C9B—S1B—O1B	−160.88 (15)
C9A—C10A—C11A—C12A	−0.3 (3)	C10B—C9B—S1B—O1B	18.58 (17)
C9B—C10B—C11B—C12B	0.3 (3)	C14B—C9B—S1B—O2B	−28.16 (17)
C10A—C11A—C12A—C13A	0.3 (3)	C10B—C9B—S1B—O2B	151.29 (15)
C10B—C11B—C12B—C13B	−0.3 (3)	C14B—C9B—S1B—N1B	85.44 (15)
C11A—C12A—C13A—C14A	0.0 (3)	C10B—C9B—S1B—N1B	−95.11 (15)

### (I) 3-Ethnyl-2-methyl-1-phenylsulfonyl-1H-indole Hydrogen-bond geometry (Å, º)

Cg2 is the centroid of the pyrrole ring N1A/C1A/C6A/C7A/C8A, Cg1 and Cg3 are the centroids of the benzene rings C1B–C6B and C1A–C6A.

**Table:**

*D*—H···*A*	*D*—H	H···*A*	*D*···*A*	*D*—H···*A*
C2*A*—H2*A*···O1*A*	0.93	2.36	2.941 (3)	121
C2*B*—H2*B*···O1*B*	0.93	2.38	2.957 (3)	120
C16*B*—H16*B*···O2*A*^i^	0.93	2.43	3.334 (3)	153
C10*A*—H10*A*···*Cg*1^ii^	0.93	2.95	3.728 (2)	142
C11*A*—H11*A*···*Cg*2^ii^	0.93	2.74	3.546 (2)	145
C16*A*—H16*A*···*Cg*3^iii^	0.93	2.88	3.699 (3)	148

Symmetry codes: (i) −*x*+1, *y*+1/2, −*z*+1; (ii) −*x*+2, *y*−1/2, −*z*+1; (iii) −*x*+1, *y*+1/2, −*z*.

### (II) 4-Phenylsulfonyl-3H,4H-cyclopenta[b]indol-1(2H)-one Crystal data

**Table:**

C_17_H_13_NO_3_S	*Z* = 4
*M**_r_* = 311.34	*F*(000) = 648
Triclinic, *P*1	*D*_x_ = 1.457 Mg m^−^^3^
Hall symbol: -P 1	Mo *K*α radiation, λ = 0.71073 Å
*a* = 9.8708 (6) Å	Cell parameters from 5869 reflections
*b* = 12.3914 (7) Å	θ = 1.6–26.5°
*c* = 13.1457 (12) Å	µ = 0.24 mm^−^^1^
α = 102.706 (3)°	*T* = 296 K
β = 96.552 (3)°	Block, white
γ = 111.989 (2)°	0.35 × 0.30 × 0.25 mm
*V* = 1419.70 (18) Å^3^	

### (II) 4-Phenylsulfonyl-3H,4H-cyclopenta[b]indol-1(2H)-one Data collection

**Table:**

Bruker Kappa APEXII CCD diffractometer	5869 independent reflections
Radiation source: fine-focus sealed tube	4993 reflections with *I* > 2σ(*I*)
Graphite monochromator	*R*_int_ = 0.028
ω & φ scans	θ_max_ = 26.5°, θ_min_ = 1.6°
Absorption correction: multi-scan (SADABS; Bruker, 2008)	*h* = −12→11
*T*_min_ = 0.919, *T*_max_ = 0.942	*k* = −15→15
20747 measured reflections	*l* = −16→16

### (II) 4-Phenylsulfonyl-3H,4H-cyclopenta[b]indol-1(2H)-one Refinement

**Table:**

Refinement on *F*^2^	Primary atom site location: structure-invariant direct methods
Least-squares matrix: full	Secondary atom site location: difference Fourier map
*R*[*F*^2^ > 2σ(*F*^2^)] = 0.038	Hydrogen site location: inferred from neighbouring sites
*wR*(*F*^2^) = 0.105	H-atom parameters constrained
*S* = 1.04	*w* = 1/[σ^2^(*F*_o_^2^) + (0.0542*P*)^2^ + 0.3752*P*] where *P* = (*F*_o_^2^ + 2*F*_c_^2^)/3
5869 reflections	(Δ/σ)_max_ < 0.001
397 parameters	Δρ_max_ = 0.22 e Å^−^^3^
0 restraints	Δρ_min_ = −0.46 e Å^−^^3^

### (II) 4-Phenylsulfonyl-3H,4H-cyclopenta[b]indol-1(2H)-one Special details

**Table:**

Geometry. All e.s.d.'s (except the e.s.d. in the dihedral angle between two l.s. planes) are estimated using the full covariance matrix. The cell e.s.d.'s are taken into account individually in the estimation of e.s.d.'s in distances, angles and torsion angles; correlations between e.s.d.'s in cell parameters are only used when they are defined by crystal symmetry. An approximate (isotropic) treatment of cell e.s.d.'s is used for estimating e.s.d.'s involving l.s. planes.
Refinement. Refinement of *F*^2^ against ALL reflections. The weighted *R*-factor *wR* and goodness of fit *S* are based on *F*^2^, conventional *R*-factors *R* are based on *F*, with *F* set to zero for negative *F*^2^. The threshold expression of *F*^2^ > σ(*F*^2^) is used only for calculating *R*-factors(gt) *etc*. and is not relevant to the choice of reflections for refinement. *R*-factors based on *F*^2^ are statistically about twice as large as those based on *F*, and *R*- factors based on ALL data will be even larger.

### (II) 4-Phenylsulfonyl-3H,4H-cyclopenta[b]indol-1(2H)-one Fractional atomic coordinates and isotropic or equivalent isotropic displacement parameters (Å^2^)

**Table:**

	*x*	*y*	*z*	*U*_iso_*/*U*_eq_	
C1A	0.27143 (17)	0.47276 (13)	0.54857 (12)	0.0410 (3)	
C1B	0.47089 (17)	0.18216 (14)	0.06379 (12)	0.0422 (3)	
C2A	0.16605 (19)	0.47310 (16)	0.46965 (14)	0.0520 (4)	
H2A	0.0730	0.4080	0.4430	0.062*	
C2B	0.3875 (2)	0.20192 (17)	−0.01653 (14)	0.0549 (4)	
H2B	0.4237	0.2740	−0.0353	0.066*	
C3A	0.2044 (2)	0.57378 (18)	0.43203 (14)	0.0571 (4)	
H3A	0.1357	0.5762	0.3789	0.069*	
C3B	0.2488 (2)	0.11042 (19)	−0.06764 (15)	0.0619 (5)	
H3B	0.1910	0.1209	−0.1224	0.074*	
C4A	0.3429 (2)	0.67144 (17)	0.47144 (14)	0.0557 (4)	
H4A	0.3656	0.7376	0.4439	0.067*	
C4B	0.1935 (2)	0.00281 (18)	−0.03917 (14)	0.0583 (4)	
H4B	0.0991	−0.0567	−0.0746	0.070*	
C5A	0.44741 (19)	0.67175 (15)	0.55092 (13)	0.0469 (4)	
H5A	0.5396	0.7379	0.5777	0.056*	
C5B	0.27612 (18)	−0.01695 (15)	0.04054 (13)	0.0484 (4)	
H5B	0.2384	−0.0890	0.0592	0.058*	
C6A	0.41249 (17)	0.57126 (13)	0.59034 (11)	0.0392 (3)	
C6B	0.41774 (17)	0.07334 (13)	0.09293 (11)	0.0397 (3)	
C7A	0.48985 (17)	0.53962 (13)	0.67042 (12)	0.0410 (3)	
C7B	0.53358 (17)	0.08518 (14)	0.17716 (12)	0.0409 (3)	
C8A	0.39943 (18)	0.42812 (13)	0.67492 (12)	0.0416 (3)	
C8B	0.64817 (17)	0.19489 (14)	0.19660 (12)	0.0414 (3)	
C9A	0.16768 (17)	0.15117 (13)	0.48222 (13)	0.0438 (3)	
C9B	0.83862 (18)	0.35206 (12)	0.03001 (12)	0.0430 (3)	
C10A	0.12748 (19)	0.14631 (16)	0.37681 (14)	0.0530 (4)	
H10A	0.0776	0.1920	0.3583	0.064*	
C10B	0.7815 (2)	0.32544 (15)	−0.07768 (14)	0.0538 (4)	
H10B	0.6897	0.3264	−0.1016	0.065*	
C11A	0.1624 (2)	0.07259 (18)	0.29923 (15)	0.0611 (5)	
H11A	0.1363	0.0685	0.2278	0.073*	
C11B	0.8642 (3)	0.29730 (16)	−0.14913 (15)	0.0642 (5)	
H11B	0.8293	0.2815	−0.2220	0.077*	
C12A	0.2354 (2)	0.00561 (15)	0.32725 (16)	0.0616 (5)	
H12A	0.2574	−0.0448	0.2745	0.074*	
C12B	0.9973 (2)	0.29264 (17)	−0.11305 (17)	0.0668 (6)	
H12B	1.0522	0.2738	−0.1616	0.080*	
C13A	0.2767 (3)	0.01193 (16)	0.43244 (18)	0.0669 (5)	
H13A	0.3274	−0.0335	0.4503	0.080*	
C13B	1.0500 (2)	0.31548 (18)	−0.00588 (17)	0.0669 (5)	
H13B	1.1387	0.3094	0.0175	0.080*	
C14A	0.2435 (2)	0.08522 (15)	0.51197 (15)	0.0577 (4)	
H14A	0.2714	0.0901	0.5834	0.069*	
C14B	0.97249 (19)	0.34730 (15)	0.06729 (14)	0.0552 (4)	
H14B	1.0093	0.3652	0.1401	0.066*	
C15A	0.6302 (2)	0.58497 (15)	0.74785 (13)	0.0503 (4)	
C15B	0.57228 (19)	0.01988 (15)	0.24752 (13)	0.0468 (4)	
C16A	0.6165 (2)	0.48606 (17)	0.80306 (15)	0.0602 (5)	
H16A	0.6269	0.5180	0.8792	0.072*	
H16B	0.6947	0.4579	0.7924	0.072*	
C16B	0.7286 (2)	0.10394 (17)	0.31390 (14)	0.0572 (4)	
H16C	0.7971	0.0655	0.3016	0.069*	
H16D	0.7271	0.1215	0.3893	0.069*	
C17A	0.4624 (2)	0.38091 (16)	0.75460 (14)	0.0542 (4)	
H17A	0.4707	0.3065	0.7206	0.065*	
H17B	0.4026	0.3669	0.8079	0.065*	
C17B	0.78018 (19)	0.22189 (16)	0.28064 (14)	0.0523 (4)	
H17C	0.7982	0.2917	0.3398	0.063*	
H17D	0.8697	0.2356	0.2522	0.063*	
N1A	0.26446 (15)	0.38410 (11)	0.60335 (10)	0.0431 (3)	
N1B	0.61497 (14)	0.25901 (12)	0.13099 (10)	0.0433 (3)	
O1A	−0.00723 (13)	0.25534 (12)	0.53947 (12)	0.0686 (4)	
O1B	0.64889 (16)	0.44484 (11)	0.07644 (11)	0.0635 (3)	
O2A	0.14420 (16)	0.21454 (12)	0.67831 (10)	0.0668 (4)	
O2B	0.83407 (15)	0.45097 (11)	0.22453 (10)	0.0605 (3)	
O3A	0.73912 (16)	0.68021 (13)	0.76796 (12)	0.0735 (4)	
O3B	0.49962 (15)	−0.08050 (11)	0.25493 (11)	0.0632 (3)	
S1A	0.12615 (5)	0.24657 (4)	0.58169 (3)	0.04932 (12)	
S1B	0.73718 (5)	0.39203 (3)	0.12199 (3)	0.04713 (12)	

### (II) 4-Phenylsulfonyl-3H,4H-cyclopenta[b]indol-1(2H)-one Atomic displacement parameters (Å^2^)

**Table:**

	*U*^11^	*U*^22^	*U*^33^	*U*^12^	*U*^13^	*U*^23^
C1A	0.0439 (8)	0.0406 (7)	0.0421 (8)	0.0219 (6)	0.0132 (6)	0.0088 (6)
C1B	0.0434 (8)	0.0484 (8)	0.0403 (8)	0.0227 (7)	0.0146 (6)	0.0136 (6)
C2A	0.0457 (9)	0.0564 (10)	0.0506 (9)	0.0246 (8)	0.0034 (7)	0.0062 (7)
C2B	0.0642 (11)	0.0614 (10)	0.0525 (9)	0.0346 (9)	0.0153 (8)	0.0252 (8)
C3A	0.0622 (11)	0.0711 (12)	0.0498 (9)	0.0417 (10)	0.0065 (8)	0.0173 (8)
C3B	0.0628 (11)	0.0791 (13)	0.0516 (10)	0.0414 (10)	0.0020 (8)	0.0172 (9)
C4A	0.0688 (11)	0.0601 (10)	0.0552 (10)	0.0380 (9)	0.0178 (9)	0.0267 (8)
C4B	0.0478 (10)	0.0657 (11)	0.0546 (10)	0.0241 (9)	0.0013 (8)	0.0077 (8)
C5A	0.0502 (9)	0.0458 (8)	0.0501 (9)	0.0221 (7)	0.0142 (7)	0.0182 (7)
C5B	0.0444 (8)	0.0485 (9)	0.0490 (9)	0.0177 (7)	0.0091 (7)	0.0109 (7)
C6A	0.0430 (8)	0.0408 (7)	0.0376 (7)	0.0213 (6)	0.0115 (6)	0.0097 (6)
C6B	0.0416 (8)	0.0438 (8)	0.0378 (7)	0.0205 (6)	0.0137 (6)	0.0120 (6)
C7A	0.0447 (8)	0.0411 (7)	0.0398 (7)	0.0199 (6)	0.0100 (6)	0.0121 (6)
C7B	0.0409 (8)	0.0438 (8)	0.0401 (7)	0.0179 (6)	0.0123 (6)	0.0134 (6)
C8A	0.0483 (8)	0.0401 (7)	0.0400 (8)	0.0216 (7)	0.0134 (6)	0.0103 (6)
C8B	0.0425 (8)	0.0447 (8)	0.0392 (7)	0.0184 (6)	0.0142 (6)	0.0130 (6)
C9A	0.0434 (8)	0.0342 (7)	0.0500 (8)	0.0108 (6)	0.0174 (7)	0.0105 (6)
C9B	0.0507 (9)	0.0310 (7)	0.0464 (8)	0.0132 (6)	0.0203 (7)	0.0109 (6)
C10A	0.0453 (9)	0.0544 (9)	0.0537 (10)	0.0193 (8)	0.0067 (7)	0.0093 (8)
C10B	0.0752 (12)	0.0466 (9)	0.0519 (9)	0.0316 (9)	0.0212 (8)	0.0221 (7)
C11A	0.0570 (10)	0.0623 (11)	0.0489 (10)	0.0166 (9)	0.0086 (8)	0.0027 (8)
C11B	0.1041 (17)	0.0489 (9)	0.0459 (9)	0.0319 (10)	0.0305 (10)	0.0177 (8)
C12A	0.0670 (11)	0.0396 (8)	0.0674 (12)	0.0131 (8)	0.0285 (9)	0.0027 (8)
C12B	0.0731 (13)	0.0489 (10)	0.0719 (13)	0.0158 (9)	0.0433 (11)	0.0075 (9)
C13A	0.0870 (14)	0.0439 (9)	0.0859 (14)	0.0360 (10)	0.0339 (12)	0.0256 (9)
C13B	0.0450 (10)	0.0636 (11)	0.0735 (13)	0.0128 (8)	0.0195 (9)	−0.0026 (9)
C14A	0.0819 (13)	0.0444 (9)	0.0598 (10)	0.0309 (9)	0.0271 (9)	0.0249 (8)
C14B	0.0431 (9)	0.0528 (9)	0.0521 (9)	0.0083 (7)	0.0127 (7)	0.0005 (7)
C15A	0.0519 (9)	0.0510 (9)	0.0461 (9)	0.0207 (8)	0.0049 (7)	0.0142 (7)
C15B	0.0500 (9)	0.0506 (9)	0.0467 (8)	0.0244 (7)	0.0143 (7)	0.0185 (7)
C16A	0.0644 (11)	0.0656 (11)	0.0548 (10)	0.0294 (9)	0.0046 (8)	0.0252 (9)
C16B	0.0551 (10)	0.0635 (11)	0.0517 (10)	0.0233 (9)	0.0022 (8)	0.0211 (8)
C17A	0.0673 (11)	0.0493 (9)	0.0537 (10)	0.0282 (8)	0.0129 (8)	0.0220 (8)
C17B	0.0444 (9)	0.0566 (10)	0.0498 (9)	0.0152 (7)	0.0058 (7)	0.0157 (8)
N1A	0.0447 (7)	0.0368 (6)	0.0466 (7)	0.0166 (5)	0.0114 (6)	0.0091 (5)
N1B	0.0432 (7)	0.0438 (7)	0.0450 (7)	0.0162 (6)	0.0137 (6)	0.0177 (5)
O1A	0.0418 (7)	0.0585 (8)	0.0975 (10)	0.0183 (6)	0.0212 (7)	0.0079 (7)
O1B	0.0782 (9)	0.0517 (7)	0.0823 (9)	0.0379 (7)	0.0373 (7)	0.0308 (6)
O2A	0.0813 (9)	0.0575 (7)	0.0601 (8)	0.0179 (7)	0.0396 (7)	0.0208 (6)
O2B	0.0677 (8)	0.0458 (6)	0.0519 (7)	0.0111 (6)	0.0213 (6)	0.0005 (5)
O3A	0.0600 (8)	0.0635 (8)	0.0764 (9)	0.0067 (7)	−0.0095 (7)	0.0261 (7)
O3B	0.0633 (8)	0.0541 (7)	0.0765 (9)	0.0214 (6)	0.0129 (7)	0.0332 (6)
S1A	0.0464 (2)	0.0416 (2)	0.0574 (2)	0.01393 (17)	0.02450 (19)	0.01016 (17)
S1B	0.0567 (2)	0.0362 (2)	0.0518 (2)	0.01897 (17)	0.02448 (19)	0.01275 (16)

### (II) 4-Phenylsulfonyl-3H,4H-cyclopenta[b]indol-1(2H)-one Geometric parameters (Å, º)

**Table:**

C1A—C2A	1.385 (2)	C10A—C11A	1.378 (3)
C1A—C6A	1.410 (2)	C10A—H10A	0.9300
C1A—N1A	1.4259 (19)	C10B—C11B	1.383 (3)
C1B—C2B	1.385 (2)	C10B—H10B	0.9300
C1B—C6B	1.408 (2)	C11A—C12A	1.366 (3)
C1B—N1B	1.428 (2)	C11A—H11A	0.9300
C2A—C3A	1.380 (3)	C11B—C12B	1.371 (3)
C2A—H2A	0.9300	C11B—H11B	0.9300
C2B—C3B	1.380 (3)	C12A—C13A	1.373 (3)
C2B—H2B	0.9300	C12A—H12A	0.9300
C3A—C4A	1.388 (3)	C12B—C13B	1.372 (3)
C3A—H3A	0.9300	C12B—H12B	0.9300
C3B—C4B	1.392 (3)	C13A—C14A	1.381 (3)
C3B—H3B	0.9300	C13A—H13A	0.9300
C4A—C5A	1.380 (2)	C13B—C14B	1.376 (2)
C4A—H4A	0.9300	C13B—H13B	0.9300
C4B—C5B	1.375 (2)	C14A—H14A	0.9300
C4B—H4B	0.9300	C14B—H14B	0.9300
C5A—C6A	1.393 (2)	C15A—O3A	1.211 (2)
C5A—H5A	0.9300	C15A—C16A	1.529 (2)
C5B—C6B	1.397 (2)	C15B—O3B	1.213 (2)
C5B—H5B	0.9300	C15B—C16B	1.522 (2)
C6A—C7A	1.438 (2)	C16A—C17A	1.532 (3)
C6B—C7B	1.438 (2)	C16A—H16A	0.9700
C7A—C8A	1.354 (2)	C16A—H16B	0.9700
C7A—C15A	1.454 (2)	C16B—C17B	1.537 (2)
C7B—C8B	1.350 (2)	C16B—H16C	0.9700
C7B—C15B	1.461 (2)	C16B—H16D	0.9700
C8A—N1A	1.379 (2)	C17A—H17A	0.9700
C8A—C17A	1.489 (2)	C17A—H17B	0.9700
C8B—N1B	1.3836 (19)	C17B—H17C	0.9700
C8B—C17B	1.488 (2)	C17B—H17D	0.9700
C9A—C10A	1.379 (2)	N1A—S1A	1.6747 (13)
C9A—C14A	1.383 (2)	N1B—S1B	1.6779 (13)
C9A—S1A	1.7552 (15)	O1A—S1A	1.4212 (14)
C9B—C10B	1.380 (2)	O1B—S1B	1.4236 (14)
C9B—C14B	1.383 (2)	O2A—S1A	1.4232 (14)
C9B—S1B	1.7591 (15)	O2B—S1B	1.4194 (13)
			
C2A—C1A—C6A	121.90 (15)	C11A—C12A—C13A	120.74 (17)
C2A—C1A—N1A	130.49 (15)	C11A—C12A—H12A	119.6
C6A—C1A—N1A	107.60 (13)	C13A—C12A—H12A	119.6
C2B—C1B—C6B	121.92 (15)	C11B—C12B—C13B	120.56 (17)
C2B—C1B—N1B	130.29 (15)	C11B—C12B—H12B	119.7
C6B—C1B—N1B	107.79 (13)	C13B—C12B—H12B	119.7
C3A—C2A—C1A	117.45 (16)	C12A—C13A—C14A	120.55 (18)
C3A—C2A—H2A	121.3	C12A—C13A—H13A	119.7
C1A—C2A—H2A	121.3	C14A—C13A—H13A	119.7
C3B—C2B—C1B	117.40 (17)	C12B—C13B—C14B	120.44 (19)
C3B—C2B—H2B	121.3	C12B—C13B—H13B	119.8
C1B—C2B—H2B	121.3	C14B—C13B—H13B	119.8
C2A—C3A—C4A	121.67 (16)	C13A—C14A—C9A	118.03 (18)
C2A—C3A—H3A	119.2	C13A—C14A—H14A	121.0
C4A—C3A—H3A	119.2	C9A—C14A—H14A	121.0
C2B—C3B—C4B	121.58 (17)	C13B—C14B—C9B	118.47 (17)
C2B—C3B—H3B	119.2	C13B—C14B—H14B	120.8
C4B—C3B—H3B	119.2	C9B—C14B—H14B	120.8
C5A—C4A—C3A	120.92 (17)	O3A—C15A—C7A	129.48 (16)
C5A—C4A—H4A	119.5	O3A—C15A—C16A	124.52 (16)
C3A—C4A—H4A	119.5	C7A—C15A—C16A	106.00 (14)
C5B—C4B—C3B	121.08 (17)	O3B—C15B—C7B	129.37 (16)
C5B—C4B—H4B	119.5	O3B—C15B—C16B	124.58 (15)
C3B—C4B—H4B	119.5	C7B—C15B—C16B	106.05 (14)
C4A—C5A—C6A	118.87 (16)	C15A—C16A—C17A	108.11 (14)
C4A—C5A—H5A	120.6	C15A—C16A—H16A	110.1
C6A—C5A—H5A	120.6	C17A—C16A—H16A	110.1
C4B—C5B—C6B	118.72 (16)	C15A—C16A—H16B	110.1
C4B—C5B—H5B	120.6	C17A—C16A—H16B	110.1
C6B—C5B—H5B	120.6	H16A—C16A—H16B	108.4
C5A—C6A—C1A	119.19 (14)	C15B—C16B—C17B	108.15 (14)
C5A—C6A—C7A	134.54 (15)	C15B—C16B—H16C	110.1
C1A—C6A—C7A	106.27 (13)	C17B—C16B—H16C	110.1
C5B—C6B—C1B	119.29 (14)	C15B—C16B—H16D	110.1
C5B—C6B—C7B	134.51 (15)	C17B—C16B—H16D	110.1
C1B—C6B—C7B	106.19 (13)	H16C—C16B—H16D	108.4
C8A—C7A—C6A	108.37 (13)	C8A—C17A—C16A	101.00 (13)
C8A—C7A—C15A	109.33 (14)	C8A—C17A—H17A	111.6
C6A—C7A—C15A	142.31 (14)	C16A—C17A—H17A	111.6
C8B—C7B—C6B	108.49 (13)	C8A—C17A—H17B	111.6
C8B—C7B—C15B	109.08 (14)	C16A—C17A—H17B	111.6
C6B—C7B—C15B	142.41 (14)	H17A—C17A—H17B	109.4
C7A—C8A—N1A	110.37 (13)	C8B—C17B—C16B	100.85 (13)
C7A—C8A—C17A	115.56 (14)	C8B—C17B—H17C	111.6
N1A—C8A—C17A	134.06 (14)	C16B—C17B—H17C	111.6
C7B—C8B—N1B	110.48 (14)	C8B—C17B—H17D	111.6
C7B—C8B—C17B	115.87 (14)	C16B—C17B—H17D	111.6
N1B—C8B—C17B	133.65 (14)	H17C—C17B—H17D	109.4
C10A—C9A—C14A	121.70 (15)	C8A—N1A—C1A	107.38 (12)
C10A—C9A—S1A	119.24 (13)	C8A—N1A—S1A	124.58 (11)
C14A—C9A—S1A	119.04 (13)	C1A—N1A—S1A	127.90 (11)
C10B—C9B—C14B	121.83 (15)	C8B—N1B—C1B	107.02 (12)
C10B—C9B—S1B	118.86 (13)	C8B—N1B—S1B	124.27 (11)
C14B—C9B—S1B	119.31 (12)	C1B—N1B—S1B	128.00 (11)
C11A—C10A—C9A	118.95 (17)	O1A—S1A—O2A	121.68 (9)
C11A—C10A—H10A	120.5	O1A—S1A—N1A	105.98 (7)
C9A—C10A—H10A	120.5	O2A—S1A—N1A	105.50 (8)
C9B—C10B—C11B	118.34 (18)	O1A—S1A—C9A	109.32 (8)
C9B—C10B—H10B	120.8	O2A—S1A—C9A	108.57 (8)
C11B—C10B—H10B	120.8	N1A—S1A—C9A	104.38 (7)
C12A—C11A—C10A	120.02 (18)	O2B—S1B—O1B	121.55 (8)
C12A—C11A—H11A	120.0	O2B—S1B—N1B	105.48 (7)
C10A—C11A—H11A	120.0	O1B—S1B—N1B	105.67 (7)
C12B—C11B—C10B	120.29 (18)	O2B—S1B—C9B	108.96 (8)
C12B—C11B—H11B	119.9	O1B—S1B—C9B	109.18 (8)
C10B—C11B—H11B	119.9	N1B—S1B—C9B	104.63 (7)
			
C6A—C1A—C2A—C3A	0.5 (2)	C8A—C7A—C15A—C16A	−0.11 (19)
N1A—C1A—C2A—C3A	179.23 (15)	C6A—C7A—C15A—C16A	179.9 (2)
C6B—C1B—C2B—C3B	0.1 (2)	C8B—C7B—C15B—O3B	179.72 (17)
N1B—C1B—C2B—C3B	−179.08 (16)	C6B—C7B—C15B—O3B	−1.9 (3)
C1A—C2A—C3A—C4A	−0.1 (3)	C8B—C7B—C15B—C16B	−0.37 (18)
C1B—C2B—C3B—C4B	0.7 (3)	C6B—C7B—C15B—C16B	177.98 (19)
C2A—C3A—C4A—C5A	−0.6 (3)	O3A—C15A—C16A—C17A	−179.90 (18)
C2B—C3B—C4B—C5B	−0.7 (3)	C7A—C15A—C16A—C17A	0.1 (2)
C3A—C4A—C5A—C6A	0.8 (3)	O3B—C15B—C16B—C17B	−179.60 (16)
C3B—C4B—C5B—C6B	0.0 (3)	C7B—C15B—C16B—C17B	0.49 (19)
C4A—C5A—C6A—C1A	−0.4 (2)	C7A—C8A—C17A—C16A	−0.07 (19)
C4A—C5A—C6A—C7A	−179.85 (16)	N1A—C8A—C17A—C16A	−178.58 (17)
C2A—C1A—C6A—C5A	−0.3 (2)	C15A—C16A—C17A—C8A	0.00 (19)
N1A—C1A—C6A—C5A	−179.22 (13)	C7B—C8B—C17B—C16B	0.19 (19)
C2A—C1A—C6A—C7A	179.30 (14)	N1B—C8B—C17B—C16B	−179.56 (16)
N1A—C1A—C6A—C7A	0.35 (16)	C15B—C16B—C17B—C8B	−0.40 (18)
C4B—C5B—C6B—C1B	0.8 (2)	C7A—C8A—N1A—C1A	1.23 (16)
C4B—C5B—C6B—C7B	−179.66 (16)	C17A—C8A—N1A—C1A	179.79 (16)
C2B—C1B—C6B—C5B	−0.9 (2)	C7A—C8A—N1A—S1A	177.23 (10)
N1B—C1B—C6B—C5B	178.48 (13)	C17A—C8A—N1A—S1A	−4.2 (2)
C2B—C1B—C6B—C7B	179.50 (14)	C2A—C1A—N1A—C8A	−179.78 (16)
N1B—C1B—C6B—C7B	−1.16 (16)	C6A—C1A—N1A—C8A	−0.95 (16)
C5A—C6A—C7A—C8A	179.87 (16)	C2A—C1A—N1A—S1A	4.4 (2)
C1A—C6A—C7A—C8A	0.39 (16)	C6A—C1A—N1A—S1A	−176.78 (10)
C5A—C6A—C7A—C15A	−0.1 (3)	C7B—C8B—N1B—C1B	−1.70 (16)
C1A—C6A—C7A—C15A	−179.6 (2)	C17B—C8B—N1B—C1B	178.06 (16)
C5B—C6B—C7B—C8B	−179.42 (16)	C7B—C8B—N1B—S1B	−172.72 (11)
C1B—C6B—C7B—C8B	0.15 (17)	C17B—C8B—N1B—S1B	7.0 (2)
C5B—C6B—C7B—C15B	2.2 (3)	C2B—C1B—N1B—C8B	−178.99 (16)
C1B—C6B—C7B—C15B	−178.21 (19)	C6B—C1B—N1B—C8B	1.75 (16)
C6A—C7A—C8A—N1A	−1.02 (17)	C2B—C1B—N1B—S1B	−8.4 (2)
C15A—C7A—C8A—N1A	178.98 (13)	C6B—C1B—N1B—S1B	172.33 (11)
C6A—C7A—C8A—C17A	−179.87 (13)	C8A—N1A—S1A—O1A	156.83 (13)
C15A—C7A—C8A—C17A	0.12 (19)	C1A—N1A—S1A—O1A	−28.01 (14)
C6B—C7B—C8B—N1B	0.98 (17)	C8A—N1A—S1A—O2A	26.56 (14)
C15B—C7B—C8B—N1B	179.92 (13)	C1A—N1A—S1A—O2A	−158.28 (13)
C6B—C7B—C8B—C17B	−178.83 (13)	C8A—N1A—S1A—C9A	−87.78 (14)
C15B—C7B—C8B—C17B	0.12 (19)	C1A—N1A—S1A—C9A	87.38 (13)
C14A—C9A—C10A—C11A	0.8 (3)	C10A—C9A—S1A—O1A	31.18 (15)
S1A—C9A—C10A—C11A	179.04 (13)	C14A—C9A—S1A—O1A	−150.53 (14)
C14B—C9B—C10B—C11B	−2.2 (2)	C10A—C9A—S1A—O2A	166.00 (13)
S1B—C9B—C10B—C11B	178.29 (12)	C14A—C9A—S1A—O2A	−15.71 (16)
C9A—C10A—C11A—C12A	0.1 (3)	C10A—C9A—S1A—N1A	−81.84 (14)
C9B—C10B—C11B—C12B	2.0 (3)	C14A—C9A—S1A—N1A	96.45 (14)
C10A—C11A—C12A—C13A	−0.9 (3)	C8B—N1B—S1B—O2B	−31.16 (14)
C10B—C11B—C12B—C13B	0.1 (3)	C1B—N1B—S1B—O2B	159.75 (13)
C11A—C12A—C13A—C14A	0.7 (3)	C8B—N1B—S1B—O1B	−161.07 (12)
C11B—C12B—C13B—C14B	−2.1 (3)	C1B—N1B—S1B—O1B	29.84 (15)
C12A—C13A—C14A—C9A	0.2 (3)	C8B—N1B—S1B—C9B	83.72 (14)
C10A—C9A—C14A—C13A	−1.0 (3)	C1B—N1B—S1B—C9B	−85.37 (14)
S1A—C9A—C14A—C13A	−179.20 (14)	C10B—C9B—S1B—O2B	−161.96 (12)
C12B—C13B—C14B—C9B	1.9 (3)	C14B—C9B—S1B—O2B	18.49 (15)
C10B—C9B—C14B—C13B	0.3 (3)	C10B—C9B—S1B—O1B	−27.11 (15)
S1B—C9B—C14B—C13B	179.80 (14)	C14B—C9B—S1B—O1B	153.34 (13)
C8A—C7A—C15A—O3A	179.85 (19)	C10B—C9B—S1B—N1B	85.62 (13)
C6A—C7A—C15A—O3A	−0.2 (4)	C14B—C9B—S1B—N1B	−93.92 (13)

### (II) 4-Phenylsulfonyl-3H,4H-cyclopenta[b]indol-1(2H)-one Hydrogen-bond geometry (Å, º)

Cg1 and Cg2 are the centroids of the benzene rings C9A–C14A and C1A–C6A.

**Table:**

*D*—H···*A*	*D*—H	H···*A*	*D*···*A*	*D*—H···*A*
C2*A*—H2*A*···O1*A*	0.93	2.44	3.007 (2)	119
C2*B*—H2*B*···O1*B*	0.93	2.44	3.010 (2)	120
C12*B*—H12*B*···O2*A*^i^	0.93	2.46	3.369 (3)	166
C5*A*—H5*A*···*Cg*1^ii^	0.93	2.65	3.550 (2)	164
C17*B*—H17*C*···*Cg*2^ii^	0.97	2.85	3.729 (2)	151

Symmetry codes: (i) *x*+1, *y*, *z*−1; (ii) −*x*+1, −*y*+1, −*z*+1.

### (III) 1-{2-[(E)-2-(5-Chloro-2-nitrophenyl)ethenyl]-1-phenylsulfonyl-1H-indol-3-yl}ethan-1-one chloroform monosolvate Crystal data

**Table:**

C_24_H_17_ClN_2_O_5_S·CHCl_3_	*Z* = 2
*M**_r_* = 600.27	*F*(000) = 612
Triclinic, *P*1	*D*_x_ = 1.535 Mg m^−^^3^
Hall symbol: -P 1	Mo *K*α radiation, λ = 0.71073 Å
*a* = 9.5856 (3) Å	Cell parameters from 4579 reflections
*b* = 11.2767 (4) Å	θ = 2.2–25.0°
*c* = 13.1782 (4) Å	µ = 0.58 mm^−^^1^
α = 104.9070 (11)°	*T* = 296 K
β = 108.2350 (9)°	Block, yellow
γ = 91.581 (1)°	0.35 × 0.30 × 0.25 mm
*V* = 1298.31 (7) Å^3^	

### (III) 1-{2-[(E)-2-(5-Chloro-2-nitrophenyl)ethenyl]-1-phenylsulfonyl-1H-indol-3-yl}ethan-1-one chloroform monosolvate Data collection

**Table:**

Bruker Kappa APEXII CCD diffractometer	4579 independent reflections
Radiation source: fine-focus sealed tube	4054 reflections with *I* > 2σ(*I*)
Graphite monochromator	*R*_int_ = 0.019
ω & φ scans	θ_max_ = 25.0°, θ_min_ = 2.2°
Absorption correction: multi-scan (SADABS; Bruker, 2008)	*h* = −11→11
*T*_min_ = 0.817, *T*_max_ = 0.866	*k* = −13→13
25757 measured reflections	*l* = −15→15

### (III) 1-{2-[(E)-2-(5-Chloro-2-nitrophenyl)ethenyl]-1-phenylsulfonyl-1H-indol-3-yl}ethan-1-one chloroform monosolvate Refinement

**Table:**

Refinement on *F*^2^	Primary atom site location: structure-invariant direct methods
Least-squares matrix: full	Secondary atom site location: difference Fourier map
*R*[*F*^2^ > 2σ(*F*^2^)] = 0.049	Hydrogen site location: inferred from neighbouring sites
*wR*(*F*^2^) = 0.136	H-atom parameters constrained
*S* = 1.05	*w* = 1/[σ^2^(*F*_o_^2^) + (0.0647*P*)^2^ + 1.2652*P*] where *P* = (*F*_o_^2^ + 2*F*_c_^2^)/3
4579 reflections	(Δ/σ)_max_ < 0.001
335 parameters	Δρ_max_ = 0.99 e Å^−^^3^
0 restraints	Δρ_min_ = −0.77 e Å^−^^3^

### (III) 1-{2-[(E)-2-(5-Chloro-2-nitrophenyl)ethenyl]-1-phenylsulfonyl-1H-indol-3-yl}ethan-1-one chloroform monosolvate Special details

**Table:**

Geometry. All e.s.d.'s (except the e.s.d. in the dihedral angle between two l.s. planes) are estimated using the full covariance matrix. The cell e.s.d.'s are taken into account individually in the estimation of e.s.d.'s in distances, angles and torsion angles; correlations between e.s.d.'s in cell parameters are only used when they are defined by crystal symmetry. An approximate (isotropic) treatment of cell e.s.d.'s is used for estimating e.s.d.'s involving l.s. planes.
Refinement. Refinement of *F*^2^ against ALL reflections. The weighted *R*-factor *wR* and goodness of fit *S* are based on *F*^2^, conventional *R*-factors *R* are based on *F*, with *F* set to zero for negative *F*^2^. The threshold expression of *F*^2^ > σ(*F*^2^) is used only for calculating *R*-factors(gt) *etc*. and is not relevant to the choice of reflections for refinement. *R*-factors based on *F*^2^ are statistically about twice as large as those based on *F*, and *R*- factors based on ALL data will be even larger.

### (III) 1-{2-[(E)-2-(5-Chloro-2-nitrophenyl)ethenyl]-1-phenylsulfonyl-1H-indol-3-yl}ethan-1-one chloroform monosolvate Fractional atomic coordinates and isotropic or equivalent isotropic displacement parameters (Å^2^)

**Table:**

	*x*	*y*	*z*	*U*_iso_*/*U*_eq_	
C1	0.7086 (3)	0.6423 (2)	0.02468 (19)	0.0363 (5)	
C2	0.8118 (3)	0.6193 (3)	−0.0308 (2)	0.0473 (6)	
H2	0.8776	0.5613	−0.0183	0.057*	
C3	0.8119 (3)	0.6861 (3)	−0.1048 (2)	0.0532 (7)	
H3	0.8811	0.6744	−0.1416	0.064*	
C4	0.7121 (3)	0.7699 (3)	−0.1258 (2)	0.0551 (7)	
H4	0.7147	0.8127	−0.1769	0.066*	
C5	0.6085 (3)	0.7915 (3)	−0.0725 (2)	0.0475 (6)	
H5	0.5402	0.8468	−0.0882	0.057*	
C6	0.6088 (3)	0.7281 (2)	0.00587 (19)	0.0363 (5)	
C7	0.5194 (3)	0.7305 (2)	0.0770 (2)	0.0358 (5)	
C8	0.5670 (3)	0.6490 (2)	0.13771 (19)	0.0344 (5)	
C9	0.9272 (3)	0.6604 (2)	0.2892 (2)	0.0396 (5)	
C10	1.0367 (3)	0.7193 (3)	0.2653 (2)	0.0511 (7)	
H10	1.0507	0.6906	0.1968	0.061*	
C11	1.1244 (3)	0.8211 (3)	0.3445 (3)	0.0575 (7)	
H11	1.1975	0.8622	0.3293	0.069*	
C12	1.1042 (3)	0.8619 (3)	0.4457 (3)	0.0587 (8)	
H12	1.1652	0.9297	0.4994	0.070*	
C13	0.9946 (4)	0.8035 (3)	0.4685 (2)	0.0610 (8)	
H13	0.9811	0.8326	0.5371	0.073*	
C14	0.9049 (3)	0.7025 (3)	0.3905 (2)	0.0503 (7)	
H14	0.8304	0.6630	0.4056	0.060*	
C15	0.4027 (3)	0.8131 (3)	0.0797 (2)	0.0456 (6)	
C16	0.2636 (3)	0.7740 (3)	0.0972 (3)	0.0602 (8)	
H16A	0.2515	0.8334	0.1598	0.090*	
H16B	0.2693	0.6945	0.1112	0.090*	
H16C	0.1806	0.7690	0.0318	0.090*	
C17	0.5195 (3)	0.6202 (2)	0.2245 (2)	0.0382 (5)	
H17	0.4982	0.5376	0.2199	0.046*	
C18	0.5051 (3)	0.7060 (2)	0.3097 (2)	0.0391 (5)	
H18	0.5314	0.7884	0.3164	0.047*	
C19	0.4498 (3)	0.6776 (2)	0.3943 (2)	0.0390 (5)	
C20	0.3520 (3)	0.5708 (3)	0.3647 (2)	0.0448 (6)	
H20	0.3261	0.5177	0.2931	0.054*	
C21	0.2930 (3)	0.5427 (3)	0.4399 (2)	0.0519 (7)	
C22	0.3259 (4)	0.6188 (3)	0.5464 (2)	0.0604 (8)	
H22	0.2855	0.5983	0.5962	0.072*	
C23	0.4196 (3)	0.7252 (3)	0.5773 (2)	0.0600 (8)	
H23	0.4420	0.7788	0.6484	0.072*	
C24	0.4811 (3)	0.7533 (3)	0.5031 (2)	0.0467 (6)	
C25	0.8075 (4)	0.0412 (3)	0.1647 (3)	0.0617 (8)	
H25	0.7224	0.0110	0.0962	0.074*	
N1	0.6811 (2)	0.58988 (18)	0.10476 (16)	0.0369 (4)	
N2	0.5838 (3)	0.8666 (3)	0.5447 (2)	0.0634 (7)	
O1	0.8948 (2)	0.46826 (18)	0.12207 (18)	0.0575 (5)	
O2	0.7418 (2)	0.46633 (17)	0.24146 (17)	0.0525 (5)	
O3	0.4195 (3)	0.9128 (2)	0.0629 (2)	0.0737 (7)	
O4	0.6820 (3)	0.8730 (3)	0.5074 (2)	0.0892 (9)	
O5	0.5675 (5)	0.9504 (3)	0.6176 (3)	0.1267 (14)	
Cl1	0.16953 (12)	0.40958 (9)	0.39577 (8)	0.0810 (3)	
Cl2	0.96400 (19)	0.00280 (16)	0.13322 (15)	0.1285 (5)	
Cl3	0.81010 (14)	0.20089 (8)	0.21320 (9)	0.0866 (3)	
Cl4	0.78435 (12)	−0.03140 (9)	0.26121 (8)	0.0817 (3)	
S1	0.81424 (7)	0.53154 (6)	0.18933 (5)	0.04142 (19)	

### (III) 1-{2-[(E)-2-(5-Chloro-2-nitrophenyl)ethenyl]-1-phenylsulfonyl-1H-indol-3-yl}ethan-1-one chloroform monosolvate Atomic displacement parameters (Å^2^)

**Table:**

	*U*^11^	*U*^22^	*U*^33^	*U*^12^	*U*^13^	*U*^23^
C1	0.0407 (12)	0.0370 (12)	0.0305 (11)	−0.0002 (10)	0.0139 (10)	0.0063 (9)
C2	0.0486 (15)	0.0550 (16)	0.0426 (14)	0.0093 (12)	0.0225 (12)	0.0115 (12)
C3	0.0511 (16)	0.0723 (19)	0.0420 (14)	0.0022 (14)	0.0261 (12)	0.0135 (13)
C4	0.0577 (17)	0.073 (2)	0.0445 (15)	0.0004 (15)	0.0214 (13)	0.0290 (14)
C5	0.0469 (14)	0.0583 (16)	0.0434 (14)	0.0057 (12)	0.0147 (12)	0.0249 (13)
C6	0.0365 (12)	0.0397 (12)	0.0313 (12)	−0.0014 (10)	0.0109 (10)	0.0087 (10)
C7	0.0357 (12)	0.0370 (12)	0.0359 (12)	0.0015 (10)	0.0131 (10)	0.0106 (10)
C8	0.0370 (12)	0.0307 (11)	0.0344 (12)	−0.0012 (9)	0.0139 (10)	0.0053 (9)
C9	0.0398 (13)	0.0424 (13)	0.0416 (13)	0.0119 (10)	0.0140 (11)	0.0191 (11)
C10	0.0478 (15)	0.0629 (18)	0.0515 (16)	0.0089 (13)	0.0246 (13)	0.0208 (14)
C11	0.0441 (15)	0.0681 (19)	0.0625 (19)	−0.0015 (14)	0.0174 (14)	0.0233 (15)
C12	0.0485 (16)	0.0659 (19)	0.0512 (17)	−0.0020 (14)	0.0067 (13)	0.0116 (14)
C13	0.0602 (18)	0.077 (2)	0.0407 (15)	0.0004 (16)	0.0149 (13)	0.0103 (14)
C14	0.0493 (15)	0.0634 (18)	0.0431 (15)	0.0029 (13)	0.0178 (12)	0.0204 (13)
C15	0.0471 (14)	0.0532 (16)	0.0409 (14)	0.0117 (12)	0.0168 (11)	0.0172 (12)
C16	0.0443 (15)	0.081 (2)	0.0624 (19)	0.0175 (15)	0.0227 (14)	0.0241 (16)
C17	0.0412 (13)	0.0372 (12)	0.0420 (13)	0.0025 (10)	0.0180 (10)	0.0162 (10)
C18	0.0396 (13)	0.0409 (13)	0.0394 (13)	0.0006 (10)	0.0158 (10)	0.0129 (11)
C19	0.0377 (12)	0.0468 (14)	0.0361 (12)	0.0068 (10)	0.0142 (10)	0.0152 (11)
C20	0.0504 (15)	0.0506 (15)	0.0378 (13)	0.0015 (12)	0.0184 (11)	0.0157 (11)
C21	0.0533 (16)	0.0619 (18)	0.0509 (16)	0.0021 (13)	0.0225 (13)	0.0278 (14)
C22	0.0656 (19)	0.083 (2)	0.0458 (16)	0.0061 (17)	0.0297 (14)	0.0277 (16)
C23	0.0609 (18)	0.085 (2)	0.0359 (14)	0.0089 (16)	0.0198 (13)	0.0149 (14)
C24	0.0439 (14)	0.0563 (16)	0.0388 (14)	0.0054 (12)	0.0133 (11)	0.0117 (12)
C25	0.082 (2)	0.0456 (16)	0.0510 (17)	0.0156 (15)	0.0141 (16)	0.0110 (13)
N1	0.0429 (11)	0.0347 (10)	0.0373 (11)	0.0060 (8)	0.0182 (9)	0.0109 (8)
N2	0.0646 (16)	0.0686 (17)	0.0474 (14)	−0.0064 (13)	0.0206 (13)	−0.0011 (13)
O1	0.0719 (13)	0.0485 (11)	0.0639 (13)	0.0291 (10)	0.0362 (11)	0.0168 (10)
O2	0.0672 (12)	0.0381 (10)	0.0651 (12)	0.0101 (9)	0.0292 (10)	0.0269 (9)
O3	0.0833 (16)	0.0643 (14)	0.1028 (19)	0.0341 (12)	0.0477 (15)	0.0496 (14)
O4	0.0786 (17)	0.0919 (19)	0.0841 (18)	−0.0295 (14)	0.0401 (15)	−0.0097 (15)
O5	0.159 (3)	0.097 (2)	0.107 (2)	−0.040 (2)	0.083 (2)	−0.0418 (19)
Cl1	0.0973 (7)	0.0836 (6)	0.0723 (6)	−0.0251 (5)	0.0390 (5)	0.0293 (5)
Cl2	0.1388 (12)	0.1330 (12)	0.1541 (13)	0.0563 (10)	0.0941 (11)	0.0482 (10)
Cl3	0.1221 (8)	0.0440 (4)	0.0838 (6)	0.0152 (5)	0.0248 (6)	0.0123 (4)
Cl4	0.1012 (7)	0.0670 (5)	0.0664 (5)	−0.0109 (5)	0.0135 (5)	0.0207 (4)
S1	0.0523 (4)	0.0331 (3)	0.0470 (4)	0.0137 (3)	0.0231 (3)	0.0160 (3)

### (III) 1-{2-[(E)-2-(5-Chloro-2-nitrophenyl)ethenyl]-1-phenylsulfonyl-1H-indol-3-yl}ethan-1-one chloroform monosolvate Geometric parameters (Å, º)

**Table:**

C1—C6	1.392 (4)	C15—C16	1.496 (4)
C1—C2	1.396 (3)	C16—H16A	0.9600
C1—N1	1.421 (3)	C16—H16B	0.9600
C2—C3	1.378 (4)	C16—H16C	0.9600
C2—H2	0.9300	C17—C18	1.327 (3)
C3—C4	1.380 (4)	C17—H17	0.9300
C3—H3	0.9300	C18—C19	1.473 (3)
C4—C5	1.378 (4)	C18—H18	0.9300
C4—H4	0.9300	C19—C20	1.396 (4)
C5—C6	1.398 (3)	C19—C24	1.401 (4)
C5—H5	0.9300	C20—C21	1.380 (4)
C6—C7	1.450 (3)	C20—H20	0.9300
C7—C8	1.366 (3)	C21—C22	1.377 (4)
C7—C15	1.479 (4)	C21—Cl1	1.736 (3)
C8—N1	1.420 (3)	C22—C23	1.369 (5)
C8—C17	1.460 (3)	C22—H22	0.9300
C9—C14	1.383 (4)	C23—C24	1.383 (4)
C9—C10	1.385 (4)	C23—H23	0.9300
C9—S1	1.748 (3)	C24—N2	1.461 (4)
C10—C11	1.376 (4)	C25—Cl2	1.716 (4)
C10—H10	0.9300	C25—Cl4	1.744 (3)
C11—C12	1.371 (4)	C25—Cl3	1.746 (3)
C11—H11	0.9300	C25—H25	0.9800
C12—C13	1.375 (5)	N1—S1	1.685 (2)
C12—H12	0.9300	N2—O4	1.199 (3)
C13—C14	1.372 (4)	N2—O5	1.214 (4)
C13—H13	0.9300	O1—S1	1.4229 (19)
C14—H14	0.9300	O2—S1	1.423 (2)
C15—O3	1.217 (4)		
			
C6—C1—C2	121.9 (2)	H16A—C16—H16B	109.5
C6—C1—N1	107.5 (2)	C15—C16—H16C	109.5
C2—C1—N1	130.6 (2)	H16A—C16—H16C	109.5
C3—C2—C1	117.1 (3)	H16B—C16—H16C	109.5
C3—C2—H2	121.5	C18—C17—C8	123.2 (2)
C1—C2—H2	121.5	C18—C17—H17	118.4
C2—C3—C4	121.7 (3)	C8—C17—H17	118.4
C2—C3—H3	119.2	C17—C18—C19	123.4 (2)
C4—C3—H3	119.2	C17—C18—H18	118.3
C5—C4—C3	121.4 (3)	C19—C18—H18	118.3
C5—C4—H4	119.3	C20—C19—C24	115.7 (2)
C3—C4—H4	119.3	C20—C19—C18	119.1 (2)
C4—C5—C6	118.3 (3)	C24—C19—C18	125.2 (2)
C4—C5—H5	120.8	C21—C20—C19	121.2 (3)
C6—C5—H5	120.8	C21—C20—H20	119.4
C1—C6—C5	119.6 (2)	C19—C20—H20	119.4
C1—C6—C7	107.9 (2)	C22—C21—C20	121.9 (3)
C5—C6—C7	132.5 (2)	C22—C21—Cl1	119.8 (2)
C8—C7—C6	107.9 (2)	C20—C21—Cl1	118.3 (2)
C8—C7—C15	129.5 (2)	C23—C22—C21	118.4 (3)
C6—C7—C15	122.5 (2)	C23—C22—H22	120.8
C7—C8—N1	108.7 (2)	C21—C22—H22	120.8
C7—C8—C17	130.2 (2)	C22—C23—C24	120.2 (3)
N1—C8—C17	121.1 (2)	C22—C23—H23	119.9
C14—C9—C10	121.1 (3)	C24—C23—H23	119.9
C14—C9—S1	119.4 (2)	C23—C24—C19	122.7 (3)
C10—C9—S1	119.5 (2)	C23—C24—N2	116.4 (3)
C11—C10—C9	118.9 (3)	C19—C24—N2	120.9 (2)
C11—C10—H10	120.6	Cl2—C25—Cl4	110.46 (18)
C9—C10—H10	120.6	Cl2—C25—Cl3	111.9 (2)
C12—C11—C10	120.2 (3)	Cl4—C25—Cl3	110.68 (18)
C12—C11—H11	119.9	Cl2—C25—H25	107.9
C10—C11—H11	119.9	Cl4—C25—H25	107.9
C11—C12—C13	120.5 (3)	Cl3—C25—H25	107.9
C11—C12—H12	119.8	C8—N1—C1	107.92 (19)
C13—C12—H12	119.8	C8—N1—S1	123.02 (16)
C14—C13—C12	120.4 (3)	C1—N1—S1	121.85 (16)
C14—C13—H13	119.8	O4—N2—O5	122.5 (3)
C12—C13—H13	119.8	O4—N2—C24	119.2 (3)
C13—C14—C9	118.9 (3)	O5—N2—C24	118.3 (3)
C13—C14—H14	120.5	O2—S1—O1	120.19 (12)
C9—C14—H14	120.5	O2—S1—N1	106.27 (11)
O3—C15—C7	118.3 (2)	O1—S1—N1	105.64 (11)
O3—C15—C16	120.2 (3)	O2—S1—C9	109.28 (12)
C7—C15—C16	121.5 (2)	O1—S1—C9	109.29 (13)
C15—C16—H16A	109.5	N1—S1—C9	105.06 (11)
C15—C16—H16B	109.5		
			
C6—C1—C2—C3	0.6 (4)	C18—C19—C20—C21	−177.6 (3)
N1—C1—C2—C3	−179.8 (3)	C19—C20—C21—C22	0.9 (5)
C1—C2—C3—C4	−1.7 (4)	C19—C20—C21—Cl1	178.8 (2)
C2—C3—C4—C5	0.7 (5)	C20—C21—C22—C23	0.2 (5)
C3—C4—C5—C6	1.4 (4)	Cl1—C21—C22—C23	−177.7 (3)
C2—C1—C6—C5	1.5 (4)	C21—C22—C23—C24	−1.2 (5)
N1—C1—C6—C5	−178.2 (2)	C22—C23—C24—C19	1.1 (5)
C2—C1—C6—C7	−179.3 (2)	C22—C23—C24—N2	−177.9 (3)
N1—C1—C6—C7	1.0 (3)	C20—C19—C24—C23	0.0 (4)
C4—C5—C6—C1	−2.4 (4)	C18—C19—C24—C23	176.4 (3)
C4—C5—C6—C7	178.6 (3)	C20—C19—C24—N2	179.0 (2)
C1—C6—C7—C8	0.8 (3)	C18—C19—C24—N2	−4.6 (4)
C5—C6—C7—C8	179.8 (3)	C7—C8—N1—C1	3.0 (3)
C1—C6—C7—C15	179.1 (2)	C17—C8—N1—C1	−176.4 (2)
C5—C6—C7—C15	−1.8 (4)	C7—C8—N1—S1	153.61 (17)
C6—C7—C8—N1	−2.3 (3)	C17—C8—N1—S1	−25.8 (3)
C15—C7—C8—N1	179.5 (2)	C6—C1—N1—C8	−2.4 (3)
C6—C7—C8—C17	177.0 (2)	C2—C1—N1—C8	178.0 (3)
C15—C7—C8—C17	−1.2 (4)	C6—C1—N1—S1	−153.47 (17)
C14—C9—C10—C11	0.3 (4)	C2—C1—N1—S1	26.9 (4)
S1—C9—C10—C11	179.7 (2)	C23—C24—N2—O4	150.3 (3)
C9—C10—C11—C12	0.8 (5)	C19—C24—N2—O4	−28.8 (4)
C10—C11—C12—C13	−1.3 (5)	C23—C24—N2—O5	−28.5 (5)
C11—C12—C13—C14	0.8 (5)	C19—C24—N2—O5	152.5 (4)
C12—C13—C14—C9	0.3 (5)	C8—N1—S1—O2	44.5 (2)
C10—C9—C14—C13	−0.8 (4)	C1—N1—S1—O2	−168.77 (18)
S1—C9—C14—C13	179.8 (2)	C8—N1—S1—O1	173.28 (19)
C8—C7—C15—O3	146.2 (3)	C1—N1—S1—O1	−40.0 (2)
C6—C7—C15—O3	−31.7 (4)	C8—N1—S1—C9	−71.2 (2)
C8—C7—C15—C16	−36.6 (4)	C1—N1—S1—C9	75.5 (2)
C6—C7—C15—C16	145.5 (3)	C14—C9—S1—O2	−17.3 (2)
C7—C8—C17—C18	−48.3 (4)	C10—C9—S1—O2	163.2 (2)
N1—C8—C17—C18	130.9 (3)	C14—C9—S1—O1	−150.7 (2)
C8—C17—C18—C19	176.7 (2)	C10—C9—S1—O1	29.9 (2)
C17—C18—C19—C20	−28.2 (4)	C14—C9—S1—N1	96.3 (2)
C17—C18—C19—C24	155.4 (3)	C10—C9—S1—N1	−83.1 (2)
C24—C19—C20—C21	−0.9 (4)		

### (III) 1-{2-[(E)-2-(5-Chloro-2-nitrophenyl)ethenyl]-1-phenylsulfonyl-1H-indol-3-yl}ethan-1-one chloroform monosolvate Hydrogen-bond geometry (Å, º)

**Table:**

*D*—H···*A*	*D*—H	H···*A*	*D*···*A*	*D*—H···*A*
C2—H2···O1	0.93	2.32	2.903 (4)	121
C22—H22···O2^i^	0.93	2.51	3.412 (4)	162
C25—H25···O3^ii^	0.98	2.49	3.283 (4)	138

Symmetry codes: (i) −*x*+1, −*y*+1, −*z*+1; (ii) −*x*+1, −*y*+1, −*z*.
